# Sub-Hertz resonance by weak measurement

**DOI:** 10.1038/s41467-020-15557-6

**Published:** 2020-04-09

**Authors:** Weizhi Qu, Shenchao Jin, Jian Sun, Liang Jiang, Jianming Wen, Yanhong Xiao

**Affiliations:** 1 Department of Physics, State Key Laboratory of Surface Physics, and Key Laboratory of Micro and Nano Photonic Structures (Ministry of Education), Fudan University, 200433 Shanghai, China; 2 Pritzker School of Molecular Engineering, University of Chicago, Chicago, IL 60637 USA; 3 Department of Physics, Kennesaw state University, Marietta, GA 30060 USA; 4 State Key Laboratory of Quantum Optics and Quantum Optics Devices, Institute of Laser Spectroscopy, Shanxi University, 030006 Taiyuan, Shanxi China

**Keywords:** Atomic and molecular interactions with photons, Magneto-optics, Quantum information, Quantum metrology

## Abstract

Weak measurement (WM) with state pre- and post-selection can amplify otherwise undetectable small signals and thus has potential in precision measurement applications. Although frequency measurements offer the hitherto highest precision due to the stable narrow atomic transitions, it remains a long-standing interest to develop new schemes to further escalate their performance. Here, we demonstrate a WM-enhanced correlation spectroscopy technique capable of narrowing the resonance linewidth down to 0.1 Hz in a room-temperature atomic vapour cell. The potential of this technique for precision measurement is demonstrated through weak magnetic-field sensing. By judiciously pre- and post-selecting frequency-modulated input and output optical states in a nearly orthogonal manner, a sensitivity of 7 fT Hz^−1/2^ at a low frequency near DC is achieved using only one laser beam with 15 µW of power. Additionally, our results extend the WM framework to a non-Hermitian Hamiltonian and shed new light on metrology and bio-magnetic field sensing.

## Abstract (web summary)

Although the possibility to use weak measurements for metrological purposes has long been known, its practicality and the effective potential advantage are debated. Here, the authors demonstrate a WM-based correlation spectroscopy technique allowing low-frequency fT-level magnetometry at room temperature.

## Introduction

Measurement is the basis for the practice of science. As a hallmark of quantum mechanics, this assumes an even more fundamental role, since the very act of measuring a system is irrevocably accompanied by a complementary disturbance. As prototypically modelled by von Neumann, the standard measurement process, in which a quantum “system” of interest is strongly coupled to an external measuring device (or “pointer”) with a small uncertainty, irreversibly collapses the system into an eigenstate of the Hamiltonian operator associated with the observable, yielding its corresponding eigenvalue. Contrary to this strong (projective) procedure, the notion of weak measurements (WMs) introduced by Aharonov et al.^1^ describes an intriguing situation where partial information is gained by feebly probing the system without undermining its initial state. Although the uncertainty in each measurement is large due to the weak perturbative nature of the information extraction, this can generally be overcome by averaging over a vast number of identically prepared states. What makes WM an interesting phenomenon is that by a post-selection on the prepared system, the weak value (WV) of an observable may lie well outside of the normal range of the measurement operator’s eigenvalues, or even become complex due to nontrivial interference effects of complex amplitudes. These peculiar features prove to be powerful for a deeper understanding of quantum paradoxes and addressing the important questions on the foundations of quantum mechanics^2–7^. Moreover, the prospect of a WV extending beyond the eigenvalue spectrum, often referred to as amplification^8^, has significant potential in metrological applications^9,10^, such as measuring weak signals by alleviating technical imperfections. Recently, this approach has garnered substantial interest and resulted in many astounding observations of birefringence effects^11^, electromagnetic pulse propagation^12^, optical spin Hall effects^13^, transverse beam deflections^14^, phase-shift time delays^15^, optical angular rotations^16^, optical frequency shift^17^, and optical nonlinearity at a few-photon level^18^, to name a few.

On the other hand, precision frequency measurements based on atomic transitions lie at the heart of many precision measurements, including atomic clocks^19^ and optical magnetometry^20^. However, a major challenge is how to attain a narrow linewidth (denoted as ${\cal{L}}$ thereafter) without sacrificing the measurement sensitivity, ascribed by the ratio of the linewidth to the signal-to-noise ratio (SNR). ${\cal{L}}$ is usually limited by the lifetime of the quantum states involved or the effective coherence time associated with the atom–light interaction, which gives the so-called natural linewidth. Note that achieving subnatural linewidths does not violate the frequency–time uncertainty relation, since the measurement time can be much longer than the coherence lifetime^21^. Although several subnatural-linewidth spectroscopy methods^19,22–27^ have been put forward in the past, most of them crucially rely on (effectively) selecting out a subgroup of atoms with a longer lifetime, and thus inevitably degrade the SNR by a larger factor than that of the ${\cal{L}}$ reduction. Even so, as emphasized by Metcalf and Phillips^22^, a narrower linewidth is still desirable, especially when unknown systematic noise deforms the lineshape. Recently, a new resonance method based on measuring the intensity-noise cross-correlations in optical fields^28–35^ has displayed the capability of reducing the resonance linewidth far below that limited by the effective coherence lifetime by 30 times^28^. Unlike other subnatural-linewidth spectroscopies^22^, this method does not diminish the sensitivity^28^ due to the absence of atom filtering, and can also resolve closely spaced multiple resonance peaks^34^. Unfortunately, to date, these demonstrations are restricted to relatively large ${\cal{L}}$ at a few kHz and beyond, which has adversely locked its potentials for precision measurements.

To overcome this barrier, here, we introduce a WM approach to the correlation spectroscopy for precise measurement of the atomic resonance by properly pre- and post-selecting the optical states. A weak coherent state of light with a modulated frequency interacts with an atom ensemble through electromagnetically induced transparency (EIT) resonance^28,32^. The underlying physical process is similar to nonlinear magneto-optical rotation (NMOR)^36^, but here the measurement is performed in a different light polarization basis. Due to the abnormal amplification induced by WVs, a sub-coherence-lifetime-limited EIT resonance linewidth down to 0.1 Hz is observed. The relation between the linewidth and the post-selection parameter is experimentally found to be linear, which is in good agreement with our theory. To demonstrate the potential applications in precision measurement, we apply this spectroscopy technique to weak magnetic field sensing. In a room temperature rubidium (Rb) vapour cell, a sensitivity of 7 fT Hz^−1/2^ is obtained in the low-frequency regime near 10–20 Hz, and less than 20 fT Hz^−1/2^ in the 2–100 Hz range. Due to the low laser power requirement (15 µW) and the long coherence lifetime enabled by anti-relaxation wall coating, the magnetometer operates close to the standard quantum limit (photon shot noise).

## Results

### Theoretical model

**Fig. 1:**
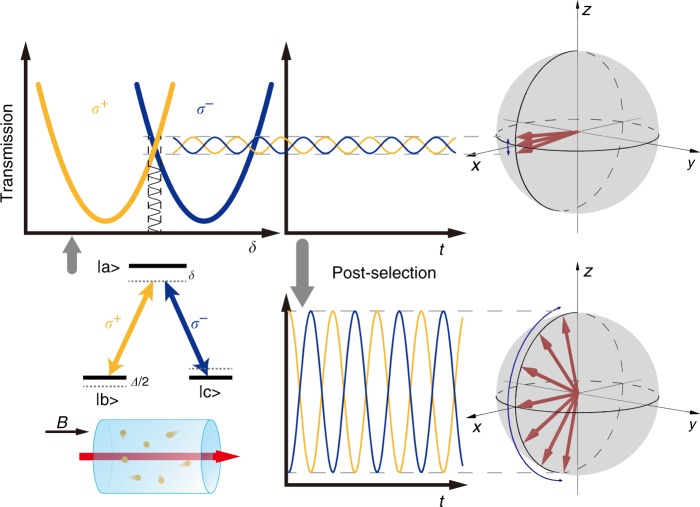
Principle and schematics of weak-measurement-based correlation spectroscopy. The *σ*^+^ and *σ*^−^ circular components of a linearly polarized laser beam form electromagnetically induced transparency (EIT) with the atoms under a three-level Λ configuration. The transmission spectra of *σ*^+^ and *σ*^−^ overlap when the two-photon-detuning *Δ* = 0, but split when *Δ* ≠ 0. The offset spectra give opposite transmission slopes for *σ*^+^ and *σ*^−^ near *δ* = 0, which are responsible for the out-of-phase FM–AM conversion and the anti-correlation. The converted intensity modulations correspond to a small oscillation of the Stokes vector of light on the Poincaré sphere, and the oscillation amplitude is proportional to *Δ*. By post-selecting the frequency components of the two transmitted EIT fields intensities, an anomalous effective amplification of the polarization oscillation can be achieved, giving rise to a reduction in the correlation resonance linewidth. Here, *δ* is the averaged one-photon detuning of the two circular light fields, and *Δ* is the two-photon detuning due to Zeeman splitting of the atomic ground states caused by the total magnetic field *B* along the light propagation direction.

Our WM protocol is realized in a generic three-level Λ-type atomic system (Fig. 1) consisting of an excited state |*a*〉 and two ground states |*b*〉 and |*c*〉, addressed by two circularly polarized laser fields *σ*^+^ (right circular) and *σ*^−^ (left circular), with amplitudes *E*_1_ and *E*_2_, respectively. The two fields are derived from one linearly polarized continuous wave (cw) laser to form EIT. The two-photon detuning *Δ* is tuned by Zeeman splitting the two ground states via a magnetic field along the light propagation direction (*B*), and the information of *B* is carried out by the transmission of the output beams. We modulate the laser frequency at angular frequency *ω*_m_, and the atom–light interaction converts this frequency modulation (FM) to amplitude (intensity) modulation (AM) for both EIT fields. The converted AM shows anti-correlations between *σ*^+^ and *σ*^−^ (see Fig. 1), when *Δ* is nonzero. As shown in Supplementary Note 1, we can derive the analytical expression of the susceptibility for the* σ*^+^ and *σ*^−^ fields from the master equation governing the atom–light interaction. The propagation effect is neglected in our model, since we have assumed an optically thin atomic medium, which is explained later in the experiment section of the manuscript. The relation between the input and output of the atomic medium of length *L* for the *σ*^+^ and *σ*^−^ fields, in terms of their Rabi frequencies Ω_in(out),r_ and Ω_in(out),l_, can be described as

1 $$\left( {\begin{array}{*{20}{c}} {{\mathrm{\Omega }}_{{\mathrm{out}},{\mathrm{r}}}} \\ {{\mathrm{\Omega }}_{{\mathrm{out}},{\mathrm{l}}}} \end{array}} \right) = \left( {\begin{array}{*{20}{c}} {{\mathrm{\Omega }}_{{\mathrm{in}},{\mathrm{r}}}} \\ {{\mathrm{\Omega }}_{{\mathrm{in}},{\mathrm{l}}}} \end{array}} \right)e^{i{{K}}L},$$

where *e*^*iΚL*^ can be treated as an evolution operator, and

2 $$K = \varsigma \left( {\begin{array}{*{20}{c}} {\frac{{\rho _{\mathrm{r}}}}{{{\mathrm{\Omega }}_{{\mathrm{in}},{\mathrm{r}}}}}} & 0 \\ 0 & {\frac{{\rho _{\mathrm{l}}}}{{{\mathrm{\Omega }}_{{\mathrm{in}},{\mathrm{l}}}}}} \end{array}} \right)$$

with *ρ*_r,l_ being the optical coherence of the atomic ensemble. *ρ*_r,l_ are proportional to the optical susceptibilities for *σ*^+^ and *σ*^−^, respectively, and are functions of the ground-state coherence (i.e., memory of the atomic spins), which is dependent on the laser power, giving rise to nonlinearity. In Eq. (2), $\varsigma = \frac{{3N\lambda ^2}}{{16\pi ^2}}{\mathrm{\Gamma }}_0$, where *N* is the atomic density, *λ* is the laser wavelength, and Γ_0_ is the spontaneous emission rate of the excited state. Following refs. ^8,13,14^, we can write an effective Hamiltonian *H* for the light with the dimension of the wave vector. However, we note that our Hamiltonian is not a single-photon Hamiltonian as in refs. ^8,11,13,14^, because in our case the optical susceptibility depends on the laser intensity, i.e., the number of photons in the coherent state. After some algebra, we have the following optical Hamiltonian:

3 $$H = - i\xi \sigma _z \otimes \left( {| - \omega _{\mathrm{m}}\rangle \langle 0| + |\omega _{\mathrm{m}}\rangle \langle 0|} \right).$$

Here, $\xi = \frac{\varsigma }{\Gamma }\frac{M}{{1 + 3M^2}}{\mathrm{Im}}\left[ {\rho _{{\mathrm{cb}}}} \right]$ is the small, perturbative, real-valued interaction strength. $M = \frac{{\lambda _{\mathrm{m}}\omega _{\mathrm{m}}}}{\Gamma }$, where Γ is the Doppler broadened linewidth of the excited state, *λ*_m_ is the modulation depth and *ω*_m_ is the modulation frequency (angular) of the laser. Im[*ρ*_cb_] is the imaginary part of the ground-state coherence *ρ*_cb_ of the atoms, which is proportional to the two-photon detuning *Δ* and is a function of the laser intensity (see Supplementary Note 1). It can be seen that the faster the modulation, the smaller the atomic response. The “pointer” operator $$\sigma _z = \left( {\begin{array}{*{20}{c}} 1 & 0 \\ 0 & { - 1} \end{array}} \right)$$ assumes an eigenvalue of +1 or −1 when acting on the corresponding *σ*^+^-eigenvector $$\left( {\begin{array}{*{20}{c}} 1 \\ 0 \end{array}} \right)$$ or the *σ*^−^-eigenvector $$\left( {\begin{array}{*{20}{c}} 0 \\ 1 \end{array}} \right)$$, respectively. By projecting onto the final “pointer” state, the expectation value 〈*σ*_*z*_〉 quantifies the intensity difference, thus the absorption difference between the transmitted *σ*^+^ and *σ*^−^ fields. The “system” operator is the frequency operator |−*ω*_m_〉〈0| + |*ω*_m_〉〈0| acting on the Hilbert space that contains three frequency components, |−*ω*_m_〉, |*ω*_m_〉 and |0〉, and is in the rotating frame defined by the modulated laser frequency. This describes the FM–AM conversion. As usual, the frequency vector here obeys the condition 〈*ω*_*i*_|*ω*_*j*_〉 = *δ*(*ω*_*i*_ − *ω*_*j*_). We note that because the second and higher harmonics are much smaller compared to the first harmonics in the AM, they are neglected in *H*. Fundamentally distinct from traditional WM Hamiltonians, the presence of “*i*” in *H* here reveals the anti-Hermitian nature of the interaction due to the differential absorption for the *σ*^+^ and *σ*^−^ fields.

Hamiltonian (3) shows that the frequency and the polarization of the light are correlated due to the atom–light interaction. For example, as shown in Fig. 1 the DC component of the transmitted *σ*^+^ and *σ*^−^ intensities are the same, which means that *σ*_*z*_’s DC part is zero for any magnetic field *B*. However, the AC component of *σ*_z_ at frequency *ω*_m_ is nonzero and proportional to *B*. This suggests that one can perform post-selection for the laser intensities in the frequency domain to enhance the contrast of the useful signal. In light of the WM procedure, the atoms are illuminated by an *x*-polarized cw laser with the initial state prepared as a product of the “pointer” and the “system” state of light: $$|{\mathrm{\Psi }}_{{\mathrm{i}}}\rangle = |{\mathrm{\Phi }}_{{\mathrm{pi}}}\rangle \otimes |{\mathrm{\Psi }}_{{\mathrm{si}}}\rangle = \frac{1}{{\sqrt 2 }}\left( {\begin{array}{*{20}{c}} 1 \\ 1 \end{array}} \right) \otimes |0\rangle$$. After traversing the atomic medium of length *L*, the final output optical state is |Ψ_f_〉 ≈ (1 − *iHL*)|Ψ_i_〉. By post-selecting the output “system” state |Ψ_sf_〉, which is nearly orthogonal to |Ψ_si_〉 = |0〉, the polarization state of the output light |Φ_pf_〉 = 〈Ψ_sf_|Ψ_f_〉 is then obtained by tracing out the “system”. Here, $|{\mathrm{\Psi }}_{{\mathrm{sf}}}\rangle = \frac{1}{{\sqrt {\left( {1 - D} \right)^2 + 2} }}\left[ {(1 - D)|0\rangle + |\omega _{\mathrm{m}}\rangle + | - \omega _{\mathrm{m}}\rangle } \right]$ is formed by the central frequency component superposed by the first-order sidebands, where *D* is the post-selection parameter and is very close to unity. Alternatively, one can show that this post-selection operation is equivalent to subtracting the majority of the DC component of the transmitted light intensities while retaining the AC components. The asymmetric forms of |Ψ_si_〉 and |Ψ_sf_〉 are also opposed to the symmetric ones often considered in previous WV-amplification experiments^9–18^. After normalization, |Φ_pf_〉 becomes

4 $$|{\mathrm{\Phi }}_{{\mathrm{pf}}}\rangle = \left( {1 - \xi LA_{\mathrm{W}}\sigma _z} \right)|{\mathrm{\Phi }}_{{\mathrm{pi}}}\rangle \approx e^{ - \xi LA_{\mathrm{W}}\sigma _z}|{\mathrm{\Phi }}_{{\mathrm{pi}}}\rangle .$$

Here, the WV associated with the system observable is defined as

5 $$A_{\mathrm{W}} = \frac{{\left\langle {{\mathrm{\Psi }}_{{\mathrm{sf}}}} \right|\left( {| - \omega _{\mathrm{m}}\rangle \langle 0| + |\omega _{\mathrm{m}}\rangle \langle 0|} \right)\left| {{\mathrm{\Psi }}_{{\mathrm{si}}}} \right\rangle }}{{\left\langle {{\mathrm{\Psi }}_{{\mathrm{sf}}}|{\mathrm{\Psi }}_{{\mathrm{si}}}} \right\rangle }} = \frac{2}{{1 - D}},$$

which is a real-valued quantity. It is worth pointing out that in spite of the real *A*_W_, the anti-Hermiticity of the interaction Hamiltonian (3) makes the results equivalent to the imaginary WV obtained from a Hermitian one. Our imaginary WV experiment scheme based on a non-Hermitian Hamiltonian is easier to realize in practice than previous ones, which often required sophisticated interferometric systems. One can now readily have the expectation value of *σ*_*z*_ for the final state |Φ_pf_〉 of the “pointer”: $\langle \sigma _z\rangle _{{\mathrm{pf}}} = \langle {\mathrm{\Phi }}_{{\mathrm{pf}}}|\sigma _{\mathrm{z}}|{\mathrm{\Phi }}_{{\mathrm{pf}}}\rangle = - 2\xi LA_{\mathrm{W}}$, indicating the WV-amplified Stokes parameter.

Now, we establish the relation between 〈*σ*_z_〉 and the WM correlation spectroscopy and derive the resonance linewidth ${\cal{L}}$. The measured quantity here is the second-order correlation function *g*^(2)^(0) between the two EIT fields’ output intensities, *I*_1_ and *I*_2_, after the post-selection (see Methods), at the zero-time lag: $g^{\left( 2 \right)}\left( 0 \right) = \langle I_1(t)I_2(t)\rangle /\sqrt {\langle I_1^2(t)\rangle \langle I_2^2(t)\rangle }$. *g*^(2)^(0) is a function of *B* (proportional to the two-photon detuning *Δ*), giving what we call the *g*^(2)^(0) resonance profile, which peaks at 1 (correlation) for *Δ* = 0, and quickly descends to −1 (anti-correlation) for nonzero *Δ*. Since the *g*^(2)^(0) value is between −1 and 1, the half-width at half-maximum (HWHM) ${\cal{L}}$ of the *g*^(2)^(0) resonance can be deduced by seeking the zero value of *g*^(2)^(0), which corresponds to equal amount of correlation and anti-correlation. Here, correlation is contributed to by the DC components in *σ*^+^ and *σ*^−^ intensities with a strength proportional to (1 − *D*), and anti-correlation comes from the AC components in the *σ*^+^ and *σ*^−^ transmission, since they are opposite in phase (see Fig. 1). Since *σ*_z_’s AC component is related to anti-correlation and is proportional to *Δ*, the anti-correlation is more pronounced for a larger *Δ*. For larger *D* values, the correlated components in the post-selected intensities decrease, thus a smaller anti-correlated component is needed to satisfy *g*^(2)^(0) = 0, indicating a smaller *Δ* and hence a reduced linewidth ${\cal{L}}$ at a larger *D*. We can also rigorously prove that 〈*I*_1_(*t*)*I*_2_(*t*)〉 is interlinked with the Stokes parameter 〈*σ*_*z*_〉 (see Supplementary Note 6) and that the condition *g*^(2)^(0) = 0 demands $\langle \sigma _z\rangle _{{\mathrm{pf}}} = \sqrt 2$. This requirement is in opposition to 〈*σ*_*z*_〉 ≤ 1 for a standard measurement. However, it turns out that the WV amplification of 〈*σ*_*z*_〉, as derived above, solves the problem. Consequently, the *g*^(2)^(0) linewidth becomes ${\cal{L}} = \frac{1}{{\sqrt 2 \left| {L\left( {\frac{{\partial \xi }}{{\partial \Delta }}} \right)A_{\mathrm{W}}} \right|}}$, suggesting the anomalous WV-induced narrowing.

When assessing the frequency resolution of the WM correlation spectroscopy, one has to take into account the SNR in addition to the linewidth ${\cal{L}}$. As will become clear, this subnatural-linewidth spectroscopy is distinct from most line-narrowing techniques, in that the linewidth reduction does not sacrifice the frequency resolving power. Under the current WM arrangement, the ultimate SNR, only limited by the photon shot noise (see Methods), follows the trend of $\frac{{2\sqrt 2 }}{{A_{\mathbf{W}}}}\sqrt {n_{{\mathrm{ph}}}}$, where *n*_ph_ is the total photon-number rate (see Supplementary Note 8). Because both ${\cal{L}}$ and SNR are inversely proportional to *A*_W_, the ultimate frequency resolving power defined by their ratio has nothing to do with *A*_W_, in accordance with the fact that classical experiments cannot breach the quantum limit. Nevertheless, the WM strategy helps to eliminate adverse effects from technical imperfections^37^.

### Experimental setup and detection scheme

In the experiment schematically shown in Fig. 1, a linearly polarized optical beam was derived from an external cavity diode laser, and then directed into a cylindrical atomic vapour cell (2 cm in diameter and 7.1 cm in length) filled with enriched ^87^Rb at room temperature (~22 °C). For such a temperature, we have experimentally verified the optical depth (OD) by measuring the transmission of the light off two-photon resonance but on one-photon resonance and found that OD ≈ 0.21, indicating an optically thin regime. The cell was housed inside a four-layer µ-metal magnetic shield to screen out the ambient field. The alkene coating^38^ on the inner wall of the glass cell allows atoms to undergo thousands of wall collisions with little demolition of their internal quantum states. The zero-power EIT HWHM is ~1 Hz (Supplementary Note 11). We note that this linewidth is dominated by decoherence from the residual magnetic field inhomogeneities and is much larger than that limited by the coating quality^38^. Inside the shield, a solenoid was used to generate a uniform *B* field along the laser’s propagation direction, and the two-photon detuning *Δ* was introduced through Zeeman splitting. The input laser, on resonance with the ^87^Rb D_1_ line (795 nm), drives the atomic transition |*F* = 2〉 → |*F*′ = 1〉 with its two circular-polarization components, *σ*^+^ and *σ*^−^, to form EIT. Their outputs were separately detected by two photodetectors with gain to analyse their fluctuating intensities. The laser frequency was modulated at an optimized modulation frequency of 3.03 kHz, with a modulation range of 250 MHz, by varying the voltage on the piezoelectric actuator of the laser cavity. The residual amplitude modulation (RAM) and laser intensity noise were suppressed by a feedback loop controlling the RF power of an acoustic-optic modulator in the light stream before the cell.

After simultaneously recording the transmitted intensities of the *σ*^+^ and *σ*^−^ fields with the photodetectors, we performed a fast Fourier transform (FFT) and digital filtering to select the frequency component at *ω*_m_, as well as the DC part. We next numerically applied an attenuation factor (1 − *D*) to the DC component, where *D* can be optimized for the best magnetometer sensitivity. Then, the two intensity signals (in the temporal domain) for *σ*^+^ and *σ*^−^, containing the AC components at *ω*_m_ and the attenuated DC component, were used to compute the value of *g*^(2)^(0). The FFT was done for each modulation period $T = \frac{{2\pi }}{{\omega _{\mathrm{m}}}}$ and one *g*^(2)^(0) data point was produced for each period. Alternatively, the amplitude and the phase of the AC components described above could be obtained using a two-channel lock-in amplifier.

### Measurement of the WM correlation resonance linewidth

**Fig. 2:**
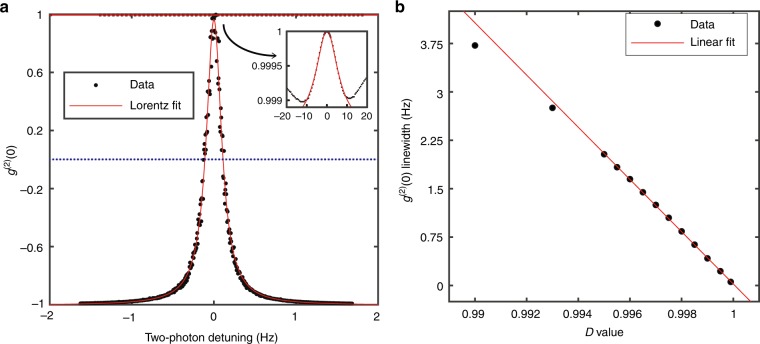
Representative correlation resonance spectrum of *g*^(2)^(0) and linewidth ${\cal{L}}$ behaviour. **a** Exemplar of the weak measurement *g*^(2)^(0) resonance spectral profile with an HWHM of approximately 0.1 Hz, smaller than the coherence-lifetime-limited HWHM of ~1 Hz, obtained by setting *D* = 0.9995. As a comparison, the inset displays the corresponding spectrum without post-selection (*D* = 0). The “two-photon detuning” of the *x-*axis is equal to the Zeeman splitting of the ground states and is proportional to the total magnetic field, including the residual stray field inside the shield and the applied magnetic field to be measured. **b** Linear dependence of the full-width at half-maximum (FWHM) of the *g*^(2)^(0) resonance on the projection parameter *D*, for an input *x*-polarized cw laser of power 15 µW, which is in good agreement with the trend predicted by our theory.

We first investigate the linewidth ${\cal{L}}$ of the *g*^(2)^(0) resonance as a function of the post-selection parameter *D*. With an optimized modulation range and laser power, we can obtain the *g*^(2)^(0) resonance profile with an HWHM of 0.1 Hz for *D* = 0.9995, as shown in Fig. 2a. This linewidth is much smaller than the coherence-lifetime-limited width of ~1 Hz, and 130 times narrower than the power-broadened EIT linewidth 13 Hz for the 15 µW total operation laser power. As one can see, the *g*^(2)^(0) resonance profile, displaying a full correlation and anti-correlation, is well fitted by a Lorentzian lineshape, as predicted by our theory. The linear dependence of ${\cal{L}}$ on *D* was also well confirmed in Fig. 2b, where ${\cal{L}}$ monotonically decreases as *D* increases. In the experiment, we found that ${\cal{L}}$ can be further reduced by increasing *D* but with a worse SNR, and the *g*^(2)^(0) resonance lineshape also deviates from a Lorentzian. Intuitively, as *D* approaches one, the correlated DC components in the output nearly vanish; while random noise, such as laser intensity noise (including RAM), becomes dominant and yields correlation signals that fluctuate, resulting in the SNR drop.

### Magnetometer results

**Fig. 3:**
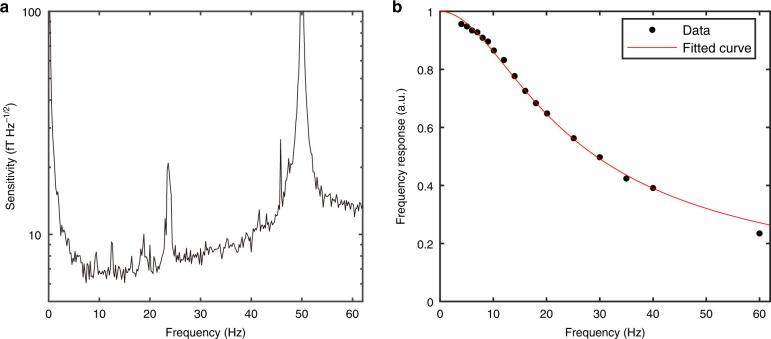
Sensitivity and frequency response of the weak measurement resonance magnetometer. **a** The sensitivity spectrum derived from the *g*^(2)^(0) noise spectrum divided by the “resonance slope vs. frequency” curve, or the response curve. The former was generated by a fast Fourier transform of 30,300 continuous data points of *g*^(2)^(0) values measured at *g*^(2)^(0) ~ 0, within 10 s, with each data point obtained from the time duration of one modulation period. **b** The normalized *g*^(2)^(0) resonance slope measured at *g*^(2)^(0) ~ 0 for different AC magnetic field frequencies, with respect to the slope measured for a DC magnetic field. The fitting curve follows the function ${\mathrm{BW}}/\sqrt {{\mathrm{BW}}^2 + f^2}$ with BW = 16 Hz. Experimental parameters: the total input laser power of 15 µW and *D* = 0.995. The test DC field we applied is 0.1 nT for assessing the magnetometer sensitivity. The magnitude of the oscillating magnetic field for measuring the atomic frequency response is 3.9 pT.

We next use this WM-correlation resonance for weak *B-*field sensing. To experimentally probe the magnetometer sensitivity, we apply a weak DC magnetic field corresponding to *g*^(2)^(0) ~ 0, where the resonance slope is large. As illustrated in Fig. 3 and in Supplementary Fig. 3, our magnetometer is sensitive to low-frequency *B* fields up to ~200 Hz. The magnetometer bandwidth is determined by the optical pumping rate for the EIT process. The best sensitivity of 7 fT Hz^−1/2^ falls in the 10–20 Hz range, with the post-selection parameter *D* = 0.995. This *D* value corresponds to an HWHM of ~1 Hz for the *g*^(2)^(0) profile, which is much smaller than the power-broadened EIT resonance linewidth of 13 Hz. The optimization of *D* for the best *B* sensitivity turns out to be a trade-off between ${\cal{L}}$ and SNR: a smaller ${\cal{L}}$ requires a larger *D*, but a larger *D* aggravates the effect of the laser intensity noise on the *g*^(2)^(0) value and results in a drastic reduction of the SNR, as explained in the previous subsection. With an overall sensitivity below 10 fT Hz^−1/2^ in the range of 5–40 Hz, and 20 fT Hz^−1/2^ up to 100 Hz (see Supplementary Note 10), this magnetometer is suitable for low-frequency *B*-field sensing. The room temperature and low laser power operation conditions make the scheme attractive for practical applications^39^.

**Fig. 4:**
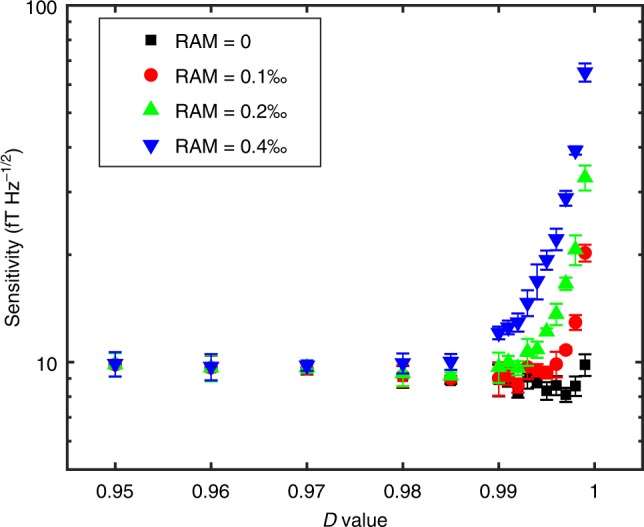
Magnetometer sensitivity versus the projection parameter *D*. Different amounts of common-mode white intensity noise were added to the output intensities to mimic RAM. The magnetic field sensitivity is the average sensitivity in the frequency range between 12 and 22 Hz with a properly chosen *D* value to optimize the sensitivity. Experimental parameters: laser power of 15 µW. The test DC field we applied is 0.1 nT to assess the magnetometer sensitivity. The error bar is the standard deviation of five repetitive experiments.

**Fig. 5:**
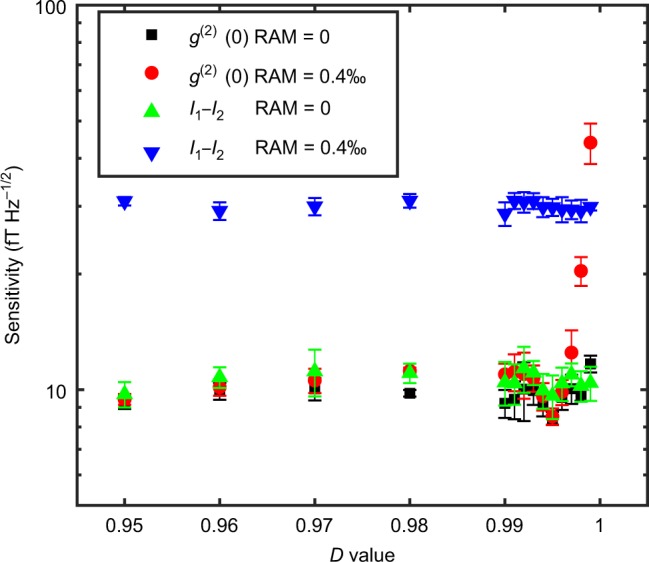
Comparison of the weak measurement resonance and the intensity-difference technique. For *D* values less than 0.998, the performance of the intensity-difference method is inferior to that of the weak measurement (WM) correlation method in the presence of residual amplitude modulation noise (RAM). Without RAM, although both methods are comparable in terms of sensitivity, the intensity-difference method experiences a power-broadened linewidth of 13 Hz, which is much wider than that attained by the WM-*g*^(2)^ process. The magnetic field sensitivity was the average sensitivity in the frequency range between 12 and 22 Hz. Experimental parameters: laser input power 15 µW with an ellipticity 1:2 in its input polarization. The error bar is the standard deviation of five repetitive experiments.

Finally, we investigate the advantages of the WM-correlation approach for magnetometry in the presence of noise, when compared to other measurement techniques. As stated above, WM cannot break the quantum limit in spite of the line-narrowing effect, but it can alleviate the adverse effects of technical noise on the sensitivity by engineering the overlap between the initial and final “system” states. First, we study how the post-selection parameter *D* affects the sensitivity. In our FM experiment, the laser intensity noise, including RAM, is a typical noise source that contributes to a positive correlation, affecting both the signal level and the noise of *g*^(2)^(0), as derived in Supplementary Note 9. To investigate its effects in a controllable way, we have designed a feedback loop to reduce the RAM level by 25 dB, and instead added a common-mode random intensity noise at the two outputs of *σ*^+^ and *σ*^−^ to mimic RAM and the laser intensity noise. Similar to previous WM work, the sensitivity becomes worse when |Ψ_si_〉 and |Ψ_sf_〉 are almost orthogonal (i.e., *D* ≈ 1), because technical noise overwhelms the signal. As shown in Fig. 4, the sensitivity degrades drastically as *D* approaches one, and it also gradually becomes worse with an increasing RAM. However, when *D* ≤ 0.98, the RAM level does not substantially affect the sensitivity, since the projected DC component in the intensity dominates over the noise. Second, we compared our scheme with the traditional lock-in detection^40^, which usually only uses the AC signal and is represented here by the intensity-difference method, i.e., taking the intensity difference of *σ*^+^ and *σ*^−^ at frequency *ω*_m_. For an impure input linear polarization along with RAM, we found that the WM-*g*^(2)^(0) method can outperform the direct intensity-difference measurement for proper *D* settings, since the WM-*g*^(2)^(0) is more immune to the RAM noise (see Fig. 5). When no RAM exists, although these two methods yield similar sensitivities, the WM-*g*^(2)^(0) scheme is still superior in that it produces a much narrower resonance linewidth for spectroscopy, whereas the intensity-difference method gives a normal power-broadened linewidth. Third, unlike NMOR, where the polarization rotation of the input light is measured, i.e., the phase difference between the two circular light components^41^, in our work, we measure the intensity difference between them. Such a measurement basis selection may become advantageous when there is a quantum backaction noise (see Methods), which is a factor preventing some magnetometers from reaching the photon-shot-noise-limited sensitivity^41^.

## Discussions

The magnetometer demonstrated here can potentially be used for biomagnetic field applications, such as cardio-signal detections^42^, if a smaller sized Cs (higher density than Rb) cell were used. To the best of our knowledge, previous fT-level low-frequency magnetometers required high-temperature (above 100 °C) operation conditions^43–45^ and this work provides such magnetometers that operate at room temperature.

The work presented also exhibits a few intriguing features beyond the existing WV works, even from a theoretical perspective. For instance, instead of choosing light polarization and propagation direction as the “system” and “pointer”, our scheme exploits optical frequency and light polarization for the “system” and “pointer”, respectively. We notice that although changing the dimensionality between the system and pointer was utilized in the direct measurement of the quantum wavefunction^46^, WV amplification is still generally absent from that method. The frequency post-selection here gives rise to WV-amplified light polarization and a substantial reduction of the resonance linewidth. Although linewidth reduction was achieved in the WM before on the dynamics of spontaneous emission in cold atoms^47^, the theoretically estimated frequency resolution is four orders of magnitude worse than our experimental results. More strikingly, the weak interaction between the system and pointer in our scheme is intrinsically non-Hermitian and characterized by a pure imaginary Hamiltonian. Consequently, counter observations are expected compared with conventional Hermitian Hamiltonians^1–18^. One practical advantage of this inverse effect is the simplicity of producing an imaginary WV without any sophisticated interferometry setup.

In short, we have used WV amplification to narrow the *g*^(2)^(0)-resonance linewidth in a room-temperature ^87^Rb vapour well below that limited by the coherence lifetime. The simplicity, low power consumption, and good sensitivity of the WM-*g*^(2)^(0)-based magnetometer makes it promising for real applications in the low-frequency magnetic field sensing. Despite the drawback of having to sacrifice the dynamical range for a detectable magnetic field, this could be overcome by using two EIT fields with a frequency offset nearly equal to twice the Larmor frequency. As a subnatural-linewidth spectroscopy method, this approach is useful for determining the resonance centre, as emphasized in ref. ^22^. Furthermore, this resonance scheme belongs to the broad category of noise correlation spectroscopy, which is deemed to hold potential in other precision measurement applications, such as detecting low-energy modes^48^ and optical forces in cold atoms^49^. Future development of this technique may incorporate quantum enhancement by including squeezed light or squeezed atomic spin^50^.

## Methods

### Non-Hermitian Hamiltonian for light

By using the atom–light interaction Hamiltonian and solving the master equation, one can obtain the susceptibility for light (see Supplementary Note 1). Following the convention that the effective Hamiltonian *H* for light is written as its wave vector^11,14^, we have arrived at the expression for *H* in Eq. (3): *H* = −*iξσ*_*z*_ ⊗ (|−*ω*_m_〉〈0| + |*ω*_m_〉〈0|), which describes the coupling between the frequency of light (“system”) and its polarization (“pointer”) denoted by the light Stokes operator *σ*_*z*_. Here, $\xi = \frac{\varsigma }{\Gamma }\frac{M}{{1 + 3M^2}}{\mathrm{Im}}\left[ {\rho _{{\mathrm{cb}}}} \right]$, with ${\mathrm{Im}}\left[ {\rho _{{\mathrm{cb}}}} \right] \propto \frac{{2\left( {1 \,+\, M^2} \right)\left( {1 \,+\, 3M^2} \right)\Gamma _{{\mathrm{p}}0}}}{{\left[ {(1 \,+\, 3M^2)\gamma _2 + 2(1 \,+\, M^2)\Gamma _{{\mathrm{p}}0}} \right]^2}}{\Delta}$, at the limit of small two-photon detuning *Δ*(≪Γ_p0_). *γ*_2_ is the dephasing rate of the ground-state coherence, and Γ_p0_ represents the optical pumping rate. Other variables are defined in the main text. Physically, this optical Hamiltonian *H* characterizes the differential absorption between the two circularly polarized EIT fields at the modulation frequency *ω*_m_. Because the system is open and the interaction is non-conservative, *H* is non-Hermitian. We note that previous WMs involving light often measured the phase of light, which is associated with a Hermitian optical Hamiltonian^11,14^. On the other hand, the differential absorption at frequency *ω*_m_ carries the information of the magnetic field *B* along the light propagation direction, since the interaction strength *ξ* in *H* is proportional to *B*. It is important to note that when the laser is on one-photon resonance and there is no modulation (*M* = 0), the absorption difference between the two EIT fields characterized by *σ*_*z*_ vanishes completely. This explains why previous atomic magnetometers usually did not measure *σ*_*z*_, and instead measured *σ*_*y*_.

### Noise correlation spectroscopy and its relation to *σ*_*z*_

In our protocol, the measured quantity is the intensity cross-correlation $g^{\left( 2 \right)}\left( 0 \right) = \langle I_1(t)I_2(t)\rangle _T/\sqrt {\langle I_1^2(t)\rangle _T\langle I_2^2(t)\rangle _T}$ between the two EIT fields’ (*σ*^+^ and *σ*^−^ components in the laser) output intensities $I_{1,2}\left( t \right) = E_{1,2}^{( - )}(t)E_{1,2}^{( + )}(t)$ at the zero-time lag, where $E_{1,2}^{( + )},E_{1,2}^{( - )}$ are the electrical field amplitudes for the positive and negative frequency components, respectively. Here, $\left\langle \cdot \right\rangle _T$ represents ensemble averaging over the time of one modulation period $T = \frac{{2\pi }}{{\omega _{\mathrm{m}}}}$. In our WM protocol, the output intensities for the post-selected *E*_1_ and *E*_2_ are respectively replaced by *I*_1_(*t*) = *I*_r_(*t*) − *D*〈*I*_r_(*t*)〉_*T*_ and *I*_2_(*t*) = *I*_l_(*t*) − *D*〈*I*_l_(*t*)〉_*T*_ for the *σ*^+^ and *σ*^−^ light, where *I*_r_(*t*) and *I*_l_(*t*) are the intensities for the *σ*^+^ and *σ*^−^ light before post-selection, respectively. Thanks to the fact that *g*^(2)^(0) is bounded between the range of −1 and 1, ${\cal{L}}$ can be deduced by simply seeking the zero-values for the numerator of *g*^(2)^(0). This means solving $\left\langle {\left[ {\frac{{I_1\left( t \right) \,-\, I_2\left( t \right)}}{{I_1\left( t \right)_T \,+\, I_2\left( t \right)_T}}} \right]^2} \right\rangle _T = 1$, where the argument on the left-hand side is essentially the Stokes parameter $\left\langle {\sigma _z} \right\rangle _{{\mathrm{pf}}}$. Based on this observation, the connection between ${\cal{L}}$ and $\left\langle {\sigma _z} \right\rangle _{{\mathrm{pf}}}$ can be rigorously established via $\frac{{I_1\left( t \right)\, -\, I_2\left( t \right)}}{{\langle I_1(t)\rangle _T \,+\, \langle I_2(t)\rangle _T}} = \frac{{I_ + \left( t \right) \,-\, I_ - \left( t \right)}}{{\left( {1 - D} \right)\left( {I_ + \left( t \right) \,+\, I_ - \left( t \right)} \right)}} = \langle \sigma _z\rangle _{{\mathrm{pf}}} \times \cos \left( {\omega _{\mathrm{m}}t} \right)$ (see Supplementary Note 6). For standard measurements 〈*σ*_z_〉 ≤ 1. However, the condition of *g*^(2)^(0) = 0 demands $\left\langle {\sigma _z} \right\rangle _{{\mathrm{pf}}} = \sqrt 2$. Interestingly, the WV amplification on $\left\langle {\sigma _z} \right\rangle _{{\mathrm{pf}}}$ solves this problem. After some algebra, one can find the *g*^(2)^(0) linewidth to be ${\cal{L}} = \frac{1}{{\sqrt 2 \left| {L\left( {\frac{{\partial \xi }}{{\partial \Delta }}} \right)A_{\mathrm{W}}} \right|}}$ (see Supplementary Note 7), which suggests anomalous WV-induced narrowing.

The physics of the line-narrowing can also be intuitively understood from Fig. 1, where the transmissions of the *σ*^+^ and *σ*^−^ components of the input laser are plotted as a function of the one-photon detuning (defined as the difference between the laser frequency and the optical transition’s frequency). When the total magnetic field *B* is zero, the two transmission spectra overlap. However, when *B* is nonzero, they will split, with their centres offset by an amount proportional to *B*^28^, resembling the nonlinear Faraday effect. Hence, when the FM is applied to the laser as shown in Fig. 1, the modulation is converted to intensity modulations in *σ*^+^ and *σ*^−^ light, which are anti-correlated due to their opposite slopes on the transmission spectra. For a relatively small *B* as considered in the current work, the slope increases along with the enlarged splitting, giving rise to intensity modulations with a larger modulation amplitude. On the other hand, the correlated part is given by the post-selected DC component (approximately independent of B, when *Δ* is much smaller than the power broadened EIT width) in the intensities. The *g*^(2)^(0) value now becomes *B*-dependent since it is determined by the competition between the anti-correlated intensity components and the correlated components. Based on this physics picture, one can now understand that the amplification of the anti-correlated component with respect to the correlated component makes *g*^(2)^(0) cross zero at a smaller two-photon detuning, giving rise to a narrowed linewidth. In previously demonstrated *g*^(2)^(0) spectroscopy, the DC component was discarded completely and the correlation part was from the AC component at 2*ω*_m_ in the intensities, which is from a small higher order effect^28^ and is hence subject to intensity noise in the laser.

### Resonance linewidth and magnetometer bandwidth

The bandwidth of a magnetometer refers to the range of frequencies of the AC magnetic field that the atoms can sense. Beyond this bandwidth, the dynamics of the atomic states cannot follow the changes in the applied magnetic field. The linewidth of the resonance is related to the system dynamics, and in many cases, it is close to the magnetometer bandwidth. However, other factors exist that can affect the linewidth, such as the measurement scheme. For example, by measuring *g*^(2)^(0), the linewidth of the resonance can be much narrower than that of the bandwidth of the system. Other line-narrowing effects include propagation effects^19^, density narrowing^51^, etc. A review of line-narrowing mechanisms in EIT can be found in ref. ^52^. Even in the absence of these line-narrowing effects, the bandwidth may not be equal to the linewidth, because the frequency response curve for the atomic magnetometer may not be Lorentzian, although the resonance spectra are often Lorentzian. For example, in our case, the EIT HWHM is 13 Hz, which is mainly determined by the optical pumping rate. However, the frequency response curve follows the form ${\mathrm{BW}}/\sqrt {{\mathrm{BW}}^2 + f^2}$ (ref. ^44^) with BW = 16 Hz, and its HWHM is 28 Hz. This again shows the difference between the resonance linewidth and the magnetometer bandwidth.

### Calibration of the magnetic field

We calibrate the magnetic field in two independent ways. In the first way, we used a commercial Hall magnetometer to measure the field inside the shield and obtained a relation between the current in the coil and the *B* field. In the second way, we compared the EIT resonance spectrum measured by sweeping the magnetic field (as in this experiment) to the EIT spectrum measured by sweeping the frequency difference between the two EIT optical fields controlled by an acoustic-optical modulator (AOM) using the experiment setup in ref. ^53^. These two methods of varying the two-photon detuning should give the same shift of the EIT spectra, which allows calibration of the magnetic field-induced Zeeman splitting by the frequency of the RF signal driving the AOM. Through this procedure, we obtained a reliable conversion between the current in the coil and the applied magnetic field, with a discrepancy of less than 10% between these two different calibration methods. This discrepancy, however, does not affect the evaluation of the magnetometer sensitivity because it contributes to a fixed (non-fluctuating) offset in the absolute value of *B*.

### Sensitivity measurement and noise analysis

To obtain the sensitivity vs. frequency curve shown in Fig. 3a, we apply a small DC magnetic field (with unavoidable noise) that corresponds to *g*^(2)^(0) ~ 0, hence a relatively large slope on the *g*^(2)^(0) resonance profile. Then, we record the intensities of the two circular light components after the vapour cell for 10 continuous seconds. As a result of the FM to AM conversion, the recorded intensities fluctuate mainly at the modulation frequency of 3.03 kHz. One *g*^(2)^(0) value is obtained for each modulation period. Therefore, in 10 s, we accumulate 30,300 data points of *g*^(2)^(0) values, which are all near zero. An FFT is then performed over those 30,300 data points, which gives the noise spectrum of *g*^(2)^(0). Next, we measure the *g*^(2)^(0) resonance slope at *g*^(2)^(0) ~ 0 for an additionally applied AC magnetic field with different frequencies. Here, the slope is defined as the oscillation amplitude of the *g*^(2)^(0) value divided by the oscillation amplitude of the applied AC magnetic field. The frequency response is obtained as shown in Fig. 3b after we normalize the slopes at all frequencies to that at DC. We then divide the *g*^(2)^(0) noise spectrum by the fitted “slope vs. frequency” curve and obtain the sensitivity spectrum in Fig. 3a. We note that the above procedure for obtaining the sensitivity spectrum has been commonly used^44^.

We have carefully carried out a noise analysis to identify various noise sources. The laser intensity noise, including the photon shot noise, is measured at the cell output when the laser is tuned far from the one-photon resonance. By comparing the intensity noise for the on and off one-photon resonance conditions, one can identify the contribution of the laser frequency noise, which is only converted to intensity noise near one-photon resonance. Similarly, the magnetic field noise can be identified by comparing the output intensity noise on and off two-photon EIT resonance, because the magnetic field noise only couples to the light intensity when on EIT resonance. Our noise analysis shows that near 10 Hz the magnetometer sensitivity is mostly limited by the magnetic field noise from both the environment and the applied field itself. Near 40 Hz, the magnetic field noise slightly decreases, and the photon shot noise starts to play a role in the obtained sensitivity. We note that the magnetic field noise could be eliminated in a gradiometer through a multiple-channel operation^44^, which is beyond the scope of this paper. Although our current coated cell experiment is not suitable for gradiometer applications due to the motional averaging of the magnetic field across the cell volume, one can use multiple coated cells with a smaller size.

The spikes in the sensitivity spectrum of Fig. 3a have different origins. The largest spike at 50 Hz is from our AC power line, and the second largest spike at ~23 Hz is due to the vibration of our optics table. Other minor spikes are from the electronics’ noise and the magnetic field noise. Inferred from the fact that the laser noise has a relatively flat spectrum in the frequency range above 30 Hz, the sensitivity degradation (Fig. 3a) at a higher frequency above 30 Hz is due to the reduced response of the atoms, as shown in Fig. 3b. Such observations can be considered independent evidence that our magnetometer performance is approaching the fundamental quantum noise limit. In contrast, in ref. ^44^, for a similar low-frequency range, even though the response curve there is similar to ours, the sensitivity spectrum is flatter because it is all dominated by the magnetic field noise. The photon shot noise-limited sensitivity in ref. ^44^ is considerably below 1 fT Hz^−1/2^, because the probe laser has much higher power.

### Standard quantum noise limit and quantum backaction noise

Since our magnetometer does not involve any quantum resources, such as squeezed light or a squeezed atomic state, its ultimate sensitivity will only be limited by the shot noise from either the light or the atoms. For our experimental conditions, the calculated photon-shot-noise-limited sensitivity is approximately 3 fT Hz^−1/2^ near 10 Hz, and 6.8 fT Hz^−1/2^ near 40 Hz due to the weakened atomic response shown in Fig. 3b. The estimated atomic projection noise (atom shot noise) limited sensitivity in this system is only 0.2 fT Hz^−1/2^ at DC. Therefore, the sensitivity here is fundamentally limited by the photon shot noise because of a relatively low laser power. In the experiment, the best sensitivity of 7 fT Hz^−1/2^ (compared to the quantum limit of 3 fT Hz^−1/2^) was obtained at about 10 Hz, limited by the magnetic field noise. Near 40 Hz, because the magnetic field noise slightly decreases, and the shot-noise-limited sensitivity is slightly worse due to a smaller atomic response, these two factors become comparable; the obtained sensitivity of ~10 fT Hz^−1/2^ at 40 Hz (to be compared to the quantum limit of 6.8 fT Hz^−1/2^) is only a factor of 1.5 away from the photon shot-noise limit.

In addition to the standard quantum noise that originated from photons or atoms, excess quantum noise may also appear in our system due to the combined resonant atom–light interaction (EIT) and the off-resonant interaction of the EIT fields with other excited state levels, as studied before^41^. Essentially, independent photon shot noises in the two EIT fields randomize the energy difference of the two ground states through an AC-stark shift produced by the off-resonant atom–light interaction, and in turn effectively cause excess quantum noise in the *B* measurement. Such noise is also called the backaction noise. As pointed out in ref. ^41^, although this process can lead to squeezed light, it adversely degrades the polarization rotation measurement in the equator plane of the Poincaré sphere. For instance, the excess quantum noise in the Stokes component *σ*_*y*_ (nearly along the anti-squeezed quadrature of light) can be as large as 10 dB above the photon shot-noise^54^, indicating a *B* sensitivity ~3.3 times worse than the shot-noise limit. However, such backaction only enters the relative phase measurement of the two circular light components and does not affect the intensity measurements as in our protocol because of a one-axis-twisting-like light squeezing process^41^. In other words, our protocol measures the polarization rotation along the line of longitude of the Poincaré sphere, which is free from such backaction noise. Though our current experiment does not appreciably suffer from this backaction noise due to the low optical depth at room temperature, it will become apparent and dominant at higher atomic densities^41^. In such cases, quantum backaction noise has to be considered, and our measurement scheme may give a better sensitivity. Investigations of magnetometers using our scheme while in the regime of quantum backaction noise dominance are underway.

## Supplementary information

Supplementary Information for Sub-Hertz resonance by weak measurement

## Data Availability

The data used in the manuscript is available upon request.
