# Supplementary material for: Sub-Hertz resonance by weak measurement
Source: Nat Commun. 2020 Apr 9;11:1752. doi: 10.1038/s41467-020-15557-6 (PMC7145818; doi:10.1038/s41467-020-15557-6)
Supplement: Supplementary file 1 — Supplementary Information for Sub-Hertz resonance by weak measurement [file 41467_2020_15557_MOESM1_ESM.pdf]

Supplementary Information for

Sub-Hertz resonance by weak measurement

Qu and Jin et al.

Supplementary Note 1. EIT with laser frequency modulation

We consider a generic three-level Λ-type EIT configuration (Fig. 1), involving a continuous wave (cw) x-polarized laser with angular frequency ω_0. Its right-circular-polarization (r) component with Rabi frequency Ω_r is near resonant with the atomic transition $|b\rangle \rightarrow |a\rangle$, while the left-circular-polarization (l) component with Rabi frequency Ω_l is near resonantly applied to $|c\rangle \rightarrow |a\rangle$. The one-photon detuning is δ. For $\Omega_\mathrm{r} = \Omega_\mathrm{l} = \Omega_\mathrm{in}$, the atomic population is equally distributed in the two ground states $|b\rangle$ and $|c\rangle$. A magnetic field along the light propagation direction (*B*) is applied to introduce the two-photon detuning Δ in the $g^{(2)}(0)$ resonance measurement.

With frequency modulation (FM), the laser frequency can be written as

$$\omega = \omega_0 + \lambda_\mathrm{m}\omega_\mathrm{m}\cos(\omega_\mathrm{m}t), \tag{1}$$

where λ_m, ω_m and $\lambda_\mathrm{m}\omega_\mathrm{m}$ are respectively the modulation depth, modulation frequency, and modulation range. In the rotating frame, the atom-light interaction can be readily computed by applying the transformation matrix

$$U = \begin{pmatrix} 1 & 0 & 0 \\ 0 & e^{-i(\omega_0 t - \lambda_\mathrm{m}\sin(\omega_\mathrm{m}t))} & 0 \\ 0 & 0 & e^{-i(\omega_0 t - \lambda_\mathrm{m}\sin(\omega_\mathrm{m}t))} \end{pmatrix} \tag{2}$$

to the normal Hamiltonian. This leads to the time-dependent one-photon detuning:

$$\delta(t) = \lambda_\mathrm{m}\omega_\mathrm{m}\cos(\omega_\mathrm{m}t). \tag{3}$$

After some algebra, the atom-light interaction Hamiltonian becomes

$$H = \hbar\begin{pmatrix} 0 & -\frac{\Omega_\mathrm{in}}{2} & -\frac{\Omega_\mathrm{in}}{2} \\ -\frac{\Omega_\mathrm{in}^*}{2} & \lambda_\mathrm{m}\omega_\mathrm{m}\cos(\omega_\mathrm{m}t) + \frac{\Delta}{2} & 0 \\ -\frac{\Omega_\mathrm{in}^*}{2} & 0 & \lambda_\mathrm{m}\omega_\mathrm{m}\cos(\omega_\mathrm{m}t) - \frac{\Delta}{2} \end{pmatrix}. \tag{4}$$

Although the FM introduces complexity to the atom-light interaction, by expanding each atomic coherence into a Fourier series $\left(\rho = \sum_n \rho^{(n)} e^{-in\omega_\mathrm{m}t}\right)$ and by matching the terms with the same order, the major master equations then take the following form:

$$in\omega_\mathrm{m}\rho_{\mathrm{r(l)}}^{(n)} = i\frac{\lambda_\mathrm{m}\omega_\mathrm{m}}{2}\left(\rho_{\mathrm{r(l)}}^{(n+1)} + \rho_{\mathrm{r(l)}}^{(n-1)}\right) - i\left(\frac{\Delta}{2} + i\frac{\Gamma}{2}\right)\rho_{\mathrm{r(l)}}^{(n)} + \frac{i}{2}\Omega_\mathrm{in}\left(\rho_{\mathrm{aa}}^{(n)} - \rho_{\mathrm{cc(bb)}}^{(n)}\right) - \frac{i}{2}\Omega_\mathrm{in}\rho_{\mathrm{bc(cb)}}^{(n)}. \tag{5}$$

From Supplementary Equation 5, the leading harmonic terms of the optical coherence can be derived as:

$$\begin{cases} \rho_{\mathrm{r(l)}}^{(0)} = \dfrac{i\Omega_\mathrm{in}\left(\rho_{\mathrm{bb(cc)}}^{(0)} + \rho_{\mathrm{cb(bc)}}^{(0)}\right)}{\Gamma}\dfrac{M^2+1}{3M^2+1}, \\ \rho_{\mathrm{r(l)}}^{(1)} = \rho_{\mathrm{r(l)}}^{(-1)} = \dfrac{-iM\rho_{\mathrm{r(l)}}^{(0)}}{1+M^2}, \end{cases} \tag{6}$$

where

$$\begin{cases} \rho_{\mathrm{cb(bc)}}^{(0)} = -\frac{(M^2+1)\frac{\Omega_{\mathrm{in}}{}^2}{2\Gamma}}{(3M^2+1)\gamma_2+2(M^2+1)\frac{\Omega_{\mathrm{in}}{}^2}{2\Gamma}}\left(1 \pm i\Delta \frac{2(3M^2+1)}{(3M^2+1)\gamma_2+2(M^2+1)\frac{\Omega_{\mathrm{in}}{}^2}{2\Gamma}}\right) \\ \rho_{\mathrm{bb(cc)}}^{(0)} = 1/2, \\ M = \frac{\lambda_{\mathrm{m}}\omega_{\mathrm{m}}}{\Gamma}, \end{cases}, \tag{7}$$

where Γ is the Doppler broadened linewidth of the excited state $|a\rangle$, and $\frac{\Omega_{\mathrm{in}}{}^2}{2\Gamma} = \Gamma_{\mathrm{p0}}$ is the optical pumping rate on one-photon resonance. Normally, the optical pumping rate Γ_{p} is dependent on one-photon detuning δ: $\Gamma_p = \frac{\Omega_{in}^2}{2\Gamma(1+(\frac{2\delta}{\Gamma})^2)}$. We note that when $\Delta \gg 2\Gamma_{\mathrm{p}} + \gamma_2$ (where γ_2 is the ground-state decoherence rate), EIT is destroyed and ρ_{bc} approaches zero. In this regime, one can utilize $\mathrm{Im}[\rho_{\mathrm{r(l)}}]$ to extract the light transmittance as $e^{-\varsigma L/\Gamma}$, where

$$\varsigma = \frac{N\mu^2}{2\lambda\varepsilon_0\hbar} = \frac{3N\lambda^2}{16\pi^2}\Gamma_0 , \tag{8}$$

where N is the atomic density, μ is the dipole moment, λ is the input laser wavelength, and Γ_0 is the spontaneous emission rate of the excited state. After passing through the atomic medium with length L, the output fields take the following simple relation with respect to the inputs, in terms of the effective wave vectors $\kappa_{\mathrm{r(l)}}$,

$$\Omega_{\mathrm{out,r(l)}} = \Omega_{\mathrm{in}} e^{i\kappa_{\mathrm{r(l)}}L} \text{ , with } \kappa_{\mathrm{r(l)}} = \frac{\varsigma}{\Omega_{\mathrm{in}}}\rho_{\mathrm{r(l)}}. \tag{9}$$

Supplementary Note 2. Jones vector representation of light and pointer-system correlation

In the WM method, we choose the polarization and frequency degrees of freedom of light as the measuring device (pointer) and observable (system), respectively. The key to the problem is representing a polarized EM field in terms of the Jones vectors. For example, let $\mathbf{E_a}(\varpi)$ represent a monochromatic, linearly polarized EM field with angular frequency ϖ, which can be described by the orthogonal right-circular and left-circular polarization components in a normalized vector form, $\mathbf{E_a}(\varpi) = \frac{(E_{\mathrm{r}}\hat{e}_{\mathrm{r}}+E_{\mathrm{l}}\hat{e}_{\mathrm{l}})}{\sqrt{|E_{\mathrm{l}}|^2+|E_{\mathrm{l}}|^2}} e^{-i\varpi t}$ with $\hat{e}_{\mathrm{r}}$ and $\hat{e}_{\mathrm{l}}$ being the unit vectors for the right-circular-polarization and left-circular-polarization bases, respectively. In the notation of Jones vectors, $\mathbf{E_a}(\varpi)$ can be simply cast as $\mathbf{E_a}(\varpi) = \frac{1}{\sqrt{|E_{\mathrm{r}}|^2+|E_{\mathrm{l}}|^2}}\begin{pmatrix} E_{\mathrm{r}} \\ E_{\mathrm{l}} \end{pmatrix} e^{-i\varpi t}$. With this notation, identifying the polarization and the frequency degrees of freedom becomes straightforward. In the WM language, we can further retype this vector as the product of the polarization and frequency states, that is, $\mathbf{E_a}(\varpi) \to |\Phi_{\mathrm{p}}\rangle \otimes |\Psi_{\mathrm{s}}\rangle = \frac{1}{\sqrt{|E_{\mathrm{r}}|^2+|E_{\mathrm{l}}|^2}}\begin{pmatrix} E_{\mathrm{r}} \\ E_{\mathrm{l}} \end{pmatrix} \otimes |0\rangle$ with $|0\rangle$ denoting $e^{-i\varpi t}$.

Note that in the Jones vector form above, the polarization and frequency degrees of freedom are separable due to the absence of the physical coupling between them. In general, however, these two degrees of freedom can be coupled with one another. When this occurs, the factorization behaviour is no longer valid. For example, let $\mathbf{E_b}(\omega)$ represent a coherently superposed EM field by two frequencies at $\varpi + \omega_1$ and $\varpi + \omega_2$,

$$\mathbf{E_b}(\omega) = \frac{1}{\sqrt{|E_\mathrm{r}|^2+|E_\mathrm{l}|^2+|E_\mathrm{r}'|^2+|E_\mathrm{l}'|^2}}\left(E_\mathrm{r}\hat{e}_\mathrm{r}e^{-i(\varpi+\omega_1)t} + E_\mathrm{l}\hat{e}_\mathrm{l}e^{-i(\varpi+\omega_1)t} + E_\mathrm{r}'\hat{e}_\mathrm{r}e^{-i(\varpi+\omega_2)t} + E_\mathrm{l}'\hat{e}_\mathrm{l}e^{-i(\varpi+\omega_2)t}\right) \tag{10}$$

In the Jones representation, the field takes the form:

$$\mathbf{E_b}(\omega) = \frac{1}{\sqrt{|E_\mathrm{r}|^2+|E_\mathrm{l}|^2+|E_\mathrm{r}'|^2+|E_\mathrm{l}'|^2}}\begin{pmatrix}E_\mathrm{r}e^{-i(\varpi+\omega_1)t} + E_\mathrm{r}'e^{-i(\varpi+\omega_2)t}\\ E_\mathrm{l}e^{-i(\varpi+\omega_1)t} + E_\mathrm{l}'e^{-i(\varpi+\omega_2)t}\end{pmatrix}$$

$$= \frac{\sqrt{|E_\mathrm{r}|^2+|E_\mathrm{l}|^2}}{\sqrt{|E_\mathrm{r}|^2+|E_\mathrm{l}|^2+|E_\mathrm{r}'|^2+|E_\mathrm{l}'|^2}}\begin{pmatrix}E_\mathrm{r}\\ E_\mathrm{l}\end{pmatrix}e^{-i(\varpi+\omega_1)t} + \frac{\sqrt{|E_\mathrm{r}'|^2+|E_\mathrm{l}'|^2}}{\sqrt{|E_\mathrm{r}|^2+|E_\mathrm{l}|^2+|E_\mathrm{r}'|^2+|E_\mathrm{l}'|^2}}\begin{pmatrix}E_\mathrm{r}'\\ E_\mathrm{l}'\end{pmatrix}e^{-i(\varpi+\omega_2)t}. \tag{11}$$

Similarly, in the WM language, this EM field can be represented as

$$\mathbf{E_b}(\omega) \to |\Phi_\mathrm{p}\rangle \otimes |\Psi_\mathrm{s}\rangle + |\Phi_\mathrm{p}'\rangle \otimes |\Psi_\mathrm{s}'\rangle$$

$$= \frac{\sqrt{|E_\mathrm{r}|^2+|E_\mathrm{l}|^2}}{\sqrt{|E_\mathrm{r}|^2+|E_\mathrm{l}|^2+|E_\mathrm{r}'|^2+|E_\mathrm{l}'|^2}}\begin{pmatrix}E_\mathrm{r}\\ E_\mathrm{l}\end{pmatrix}\otimes|\omega_1\rangle + \frac{\sqrt{|E_\mathrm{r}'|^2+|E_\mathrm{l}'|^2}}{\sqrt{|E_\mathrm{r}|^2+|E_\mathrm{l}|^2+|E_\mathrm{r}'|^2+|E_\mathrm{l}'|^2}}\begin{pmatrix}E_\mathrm{r}'\\ E_\mathrm{l}'\end{pmatrix}\otimes|\omega_2\rangle. \tag{12}$$

With this form of the electric field, it is apparent that the polarization and frequency degrees of freedom now become non-separable, implying the formation of a classical analogue of an entangled state [1]. In our case, we have a polarization vector space and a frequency vector space, respectively, represented by the polarization Poincaré sphere and harmonic frequencies, and the classical analogue of entanglement is between these two degrees of freedom of a single system (i.e., intra-system correlation). The frequency states obey the following operations, $\langle\omega_{1,2}| = e^{i(\varpi+\omega_{1,2})t}$, and $|\omega_2\rangle\langle\omega_1| = e^{i(\omega_1-\omega_2)t}$.

Back to our experiment, the input x-polarized cw laser field takes the full initial state as

$$|\Psi_\mathrm{i}\rangle = |\Phi_\mathrm{pi}\rangle \otimes |\Psi_\mathrm{si}\rangle = \frac{1}{\sqrt{2}}\begin{pmatrix}1\\1\end{pmatrix}\otimes|0\rangle, \tag{13}$$

before the atom-light interaction. The application of FM creates a variety of new harmonic components $\pm n\omega_\mathrm{m}$ ($n \in$ integer) away from ω_0. In the Jones notation, the EM field now turns out to be $\frac{1}{\sqrt{|E_\mathrm{r}|^2+|E_\mathrm{l}|^2}}\begin{pmatrix}E_\mathrm{r}\\ E_\mathrm{l}\end{pmatrix}e^{-i[\omega_0+\lambda_\mathrm{m}\,\omega_\mathrm{m}\cos(\omega_\mathrm{m}t)]t}$. At the output, we only post-selected intensities associated with the DC component (modelled by the projection operator $\sum_{n=0}^{\infty}|n\omega_\mathrm{m}\rangle\langle n\omega_\mathrm{m}|$) and the leading AC components $\pm\omega_\mathrm{m}$ (modelled by the projection operators $\sum_{n=0}^{\infty}|(n+1)\omega_\mathrm{m}\rangle\langle n\omega_\mathrm{m}|$ and $\sum_{n=0}^{\infty}|(n-1)\omega_\mathrm{m}\rangle\langle n\omega_\mathrm{m}|$) through frequency-dependent intensity filtering. After carefully examining the weight of each frequency component in the converted AM, the contributions from these three projection operators, $|0\rangle\langle 0|$, $|\pm\omega_\mathrm{m}\rangle\langle 0|$, and $|0\rangle\langle\pm\omega_\mathrm{m}|$, are proven to be dominant both theoretically and in the measured data. Throughout the work, therefore, the truncated frequency Hilbert space (spanned by $|0\rangle$, $|\omega_\mathrm{m}\rangle$ and $|-\omega_\mathrm{m}\rangle$) will be of interest, while higher frequency components will not be taken into account. The post-selection on the frequency results in the post-selected system state as

$$|\Psi_{\mathrm{sf}}\rangle = \frac{1}{\sqrt{(1-D)^2+2}}[(1-D)|0\rangle + |\omega_{\mathrm{m}}\rangle + |-\omega_{\mathrm{m}}\rangle], \tag{14}$$

where the parameter D close to unity ascribes the DC intensity filtering in the measurement. It can be proven that such a state selection on the electrical field of light is equivalent to keeping $(1-D)$ times the DC part plus all the AC part at ω_{m} in the light intensity.

Supplementary Note 3. Stokes parameters and the "pointer" observable

The coherency matrix is a general description of light polarization. In our case, a 2 x 2 polarization coherency matrix ϱ can be attained that encodes all the available information about the light polarization,

$$\boldsymbol{\varrho} = \mathbf{E}_{\mathrm{p}}\mathbf{E}_{\mathrm{p}}^{\dagger} \text{ with } \mathbf{E}_{\mathrm{p}} = \begin{pmatrix} E_{\mathrm{r}}/\sqrt{E_{\mathrm{r}}^2+E_{\mathrm{l}}^2} \\ E_{\mathrm{l}}/\sqrt{E_{\mathrm{r}}^2+E_{\mathrm{l}}^2} \end{pmatrix}. \tag{15}$$

Alternatively, $\boldsymbol{\varrho}$ in Supplementary Equation 15 admits a Liouville representation of the form

$$\boldsymbol{\varrho} = \textstyle\sum_{j=0}^{3} S_j \boldsymbol{\sigma}_j \ , \tag{16}$$

where the coefficients S_j are the Stokes parameters, and the set $\{\boldsymbol{\sigma}_j\}_{j=0}^{3}$ forms the complete basis of observables. In polarization optics, $\{\boldsymbol{\sigma}_j\}_{j=0}^{3}$ is composed by the four Pauli matrices,

$$\boldsymbol{\sigma}_0 = \begin{pmatrix} 1 & 0 \\ 0 & 1 \end{pmatrix}, \boldsymbol{\sigma}_x = \begin{pmatrix} 0 & 1 \\ 1 & 0 \end{pmatrix}, \boldsymbol{\sigma}_y = \begin{pmatrix} 0 & -i \\ i & 0 \end{pmatrix}, \boldsymbol{\sigma}_z = \begin{pmatrix} 1 & 0 \\ 0 & -1 \end{pmatrix}. \tag{17}$$

Obviously, these are orthogonal to one another with respect to $\mathrm{Trace}(\boldsymbol{\sigma}_i\boldsymbol{\sigma}_j) = 2\delta_{ij}$. With Supplementary Equation 16 and Supplementary Equation 17 it is not difficult to show

$$S_j = \mathrm{Trace}(\boldsymbol{\varrho}\boldsymbol{\sigma}_j). \tag{18}$$

Supplementary Equation 18 thus formally provides a way to describe the Stokes parameter in terms of the pointer operator $\boldsymbol{\sigma}_j$. As an example, let us look at S_z for the x-polarized beam $|\Phi_{\mathrm{pi}}\rangle = \frac{1}{\sqrt{2}}\binom{1}{1}$. According to Supplementary Equation 18, $S_z = \langle\Phi_{\mathrm{pi}}|\boldsymbol{\sigma}_z|\Phi_{\mathrm{pi}}\rangle = 0$, as expected.

Supplementary Note 4. Physical meaning of $\langle\sigma_z\rangle$ and $\langle\sigma_z\rangle_{\mathrm{pf}}$

Since the measured $g^{(2)}(0)$ linewidth highly depends on the expectation value $\langle\sigma_z\rangle$, it would thus be interesting to look closely at this quantity with the notations defined above. We first start with the usual measurement without post-selection. For the electric field $\mathbf{E_b}(\omega)$, let us assume the weight of $|\omega_1\rangle$ is more prominent for the input x-polarized electric field. We then have

$S_z = \langle \sigma_z \rangle$

$$= \frac{(E_r e^{-i\omega_1 t} + E_r' e^{-i\omega_2 t})^*(E_r e^{-i\omega_1 t} + E_r' e^{-i\omega_2 t}) - (E_l e^{-i\omega_1 t} + E_l' e^{-i\omega_2 t})^*(E_l e^{-i\omega_1 t} + E_l' e^{-i\omega_2 t})}{(E_r e^{-i\omega_1 t} + E_r' e^{-i\omega_2 t})^*(E_r e^{-i\omega_1 t} + E_r' e^{-i\omega_2 t}) + (E_l e^{-i\omega_1 t} + E_l' e^{-i\omega_2 t})^*(E_l e^{-i\omega_1 t} + E_l' e^{-i\omega_2 t})}$$

$$= \frac{|E_r|^2 - |E_l|^2 + |E_r'|^2 - |E_l'|^2 + E_r{}^* E_r' e^{i(\omega_1-\omega_2)t} + E_r E_r{}'^* e^{-i(\omega_1-\omega_2)t} - E_l{}^* E_l' e^{i(\omega_1-\omega_2)t} - E_l E_l{}'^* e^{-i(\omega_1-\omega_2)t}}{|E_r|^2 + |E_l|^2 + |E_r'|^2 + |E_l'|^2 + E_r{}^* E_r' e^{i(\omega_1-\omega_2)t} + E_r E_r{}'^* e^{-i(\omega_1-\omega_2)t} + E_l{}^* E_l' e^{i(\omega_1-\omega_2)t} + E_l E_l{}'^* e^{-i(\omega_1-\omega_2)t}}$$

$$= \frac{|E_r|^2 - |E_l|^2 + |E_r'|^2 - |E_l'|^2 + 2(Re(E_r{}^* E_r') - Re(E_l{}^* E_l'))\cos(\omega_1-\omega_2)t}{|E_r|^2 + |E_l|^2 + |E_r'|^2 + |E_l'|^2 + 2\big(Re(E_r{}^* E_r') + Re(E_l{}^* E_l')\big)\cos(\omega_1-\omega_2)t}$$

$$\simeq \frac{2\big(Re(E_r{}^* E_r') - Re(E_l{}^* E_l')\big)\cos(\omega_1-\omega_2)t}{|E_r|^2 + |E_l|^2 + |E_r'|^2 + |E_l'|^2}. \qquad (19)$$

Assuming the post-selected frequency state is $|\Psi_{sf}\rangle = c_1|\omega_1\rangle + c_2|\omega_2\rangle$ with the normalization $|c_1|^2 + |c_2|^2 = 1$, the Stokes parameter S_z for $\mathbf{E_b}(\omega)$ now becomes

$$S_z \simeq \frac{2\big(Re(c_1{}^* c_2 E_r{}^* E_r') - Re(c_1{}^* c_2 E_l{}^* E_l')\big)}{|c_1 E_r|^2 + |c_1 E_l|^2 + |c_2 E_r'|^2 + |c_2 E_l'|^2}\cos(\omega_1-\omega_2)t. \qquad (20)$$

thus revealing how the post-selection on the frequency alters the polarization. In contrast, $\langle \sigma_z \rangle_{pf}$ represents the expectation value after the post-selection in the frequency domain, where the subscript "p" means "pointer" and "f" means "final". Again, let's assume that the weight of $|\omega_1\rangle$ is more prominent for the input x-polarized electric field. We then have

$\langle \sigma_z \rangle_{pf}$

$$= \big(\langle \Phi_p| \otimes \langle \Psi_s| + \langle \Phi_p'| \otimes \langle \Psi_s'|\big)|\Psi_{sf}\rangle \boldsymbol{\sigma}_z \langle \Psi_{sf}|\big(|\Phi_p\rangle \otimes |\Psi_s\rangle + |\Phi_p'\rangle \otimes |\Psi_s'\rangle\big)$$

$$= \frac{(c_1 E_r + c_2 E_r')^*(c_1 E_r + c_2 E_r') - (c_1 E_l + c_2 E_l')^*(c_1 E_l + c_2 E_l')}{|c_1 E_r|^2 + |c_2 E_r'|^2 + |c_1 E_l|^2 + |c_2 E_l'|^2}$$

$$\approx \frac{c_1{}^* c_2 E_r^* E_r' + c_1 c_2{}^* E_r E_r'^* - c_1{}^* c_2 E_l^* E_l' - c_1 c_2{}^* E_l E_l'^*}{|c_1 E_r|^2 + |c_2 E_r'|^2 + |c_1 E_l|^2 + |c_2 E_l'|^2}$$

$$= \frac{2\big(Re(c_1{}^* c_2 E_r{}^* E_r') - Re(c_1{}^* c_2 E_l{}^* E_l')\big)}{|c_1 E_r|^2 + |c_2 E_r'|^2 + |c_1 E_l|^2 + |c_2 E_l'|^2}, \qquad (21)$$

which is precisely the coefficient of $\cos(\omega_1-\omega_2)t$ in Supplementary Equation 20.

Then, we generalize the above derivation to the field containing three frequency components: $\begin{pmatrix} E_{r,\omega_m} \\ E_{l,\omega_m} \end{pmatrix} e^{-i\omega_m t} + \begin{pmatrix} E_{r,-\omega_m} \\ E_{l,-\omega_m} \end{pmatrix} e^{i\omega_m t} + \begin{pmatrix} E_{r,0} \\ E_{l,0} \end{pmatrix}$ as in Supplementary Equation 14: $|\Psi_{sf}\rangle = \frac{1}{\sqrt{(1-D)^2+2}}[(1-D)|0\rangle + |\omega_m\rangle + |-\omega_m\rangle]$, under the condition of $\begin{cases} E_{r,0} \gg E_{r,\omega_m}, E_{r,-\omega_m} \\ E_{l,0} \gg E_{l,\omega_m}, E_{l,-\omega_m} \end{cases}$ and $E_{r,0} \simeq E_{l,0}$.

We can obtain

$$\begin{cases} S_z \simeq \frac{2\left(Re(E_{\mathrm{r},0}{}^{*}E'_{\mathrm{r},\omega_\mathrm{m}})+Re(E_{\mathrm{r},0}{}^{*}E'_{\mathrm{r},-\omega_\mathrm{m}})-Re(E_{\mathrm{l},0}{}^{*}E'_{\mathrm{l},\omega_\mathrm{m}})-Re(E_{\mathrm{l},0}{}^{*}E'_{\mathrm{l},-\omega_\mathrm{m}})\right)}{(1-D)(|E_{\mathrm{r},0}|^2+|E_{\mathrm{l},0}|^2)}\cos(\omega_\mathrm{m})t \\ \langle\sigma_z\rangle_{\mathrm{pf}} \simeq \frac{2\left(Re(E_{\mathrm{r},0}{}^{*}E'_{\mathrm{r},\omega_\mathrm{m}})+Re(E_{\mathrm{r},0}{}^{*}E'_{\mathrm{r},-\omega_\mathrm{m}})-Re(E_{\mathrm{l},0}{}^{*}E'_{\mathrm{l},\omega_\mathrm{m}})-Re(E_{\mathrm{l},0}{}^{*}E'_{\mathrm{l},-\omega_\mathrm{m}})\right)}{(1-D)(|E_{\mathrm{r},0}|^2+|E_{\mathrm{l},0}|^2)} \end{cases}. \tag{22}$$

Again, we have

$$S_z = \langle\sigma_z\rangle_{\mathrm{pf}}\cos(\omega_\mathrm{m}t). \tag{23}$$

The underlying physics here is that the post-selection is equivalent to Fourier decomposition on $\langle\boldsymbol{\sigma}_z\rangle$ and picking up the terms only associated with the frequency component ω_m.

Supplementary Note 5. Characterization of S_z

After interacting with the atoms, the polarization of the total output light without post-selection can be evaluated by

$$S_{z,\mathrm{out}} = \frac{|\Omega_{\mathrm{out,r}}|^2-|\Omega_{\mathrm{out,l}}|^2}{|\Omega_{\mathrm{out,r}}|^2+|\Omega_{\mathrm{out,l}}|^2}. \tag{24}$$

Using Supplementary Equation 9, we can represent $S_{z,\mathrm{out}}$ (Supplementary Equation 24) in terms of the atomic coherences:

$$S_{z,\mathrm{out}} = \frac{e^{-\frac{2\varsigma L}{\Omega_\mathrm{in}}\mathrm{Im}[\rho_\mathrm{r}]}-e^{-\frac{2\varsigma L}{\Omega_\mathrm{in}}\mathrm{Im}[\rho_\mathrm{l}]}}{e^{-\frac{2\varsigma L}{\Omega_\mathrm{in}}\mathrm{Im}[\rho_\mathrm{r}]}+e^{-\frac{2\varsigma L}{\Omega_\mathrm{in}}\mathrm{Im}[\rho_\mathrm{l}]}}, \tag{25}$$

which provides a connection between the output $S_{z,\mathrm{out}}$ and the atomic coherences $\rho_{\mathrm{r,l}}$.
Under the condition of $\varsigma L \ll \Gamma$, Supplementary Equation 25 is simplified as

$$S_{z,\mathrm{out}} = \frac{\varsigma L\Omega_\mathrm{in}\mathrm{Im}[\rho_\mathrm{r}-\rho_\mathrm{l}]}{|\Omega_\mathrm{in}|^2-\varsigma L\Omega_\mathrm{in}\mathrm{Im}[\rho_\mathrm{r}+\rho_\mathrm{l}]}. \tag{26}$$

The application of Supplementary Equations 6 -7 gives

$$\begin{cases} \mathrm{Im}[\rho_\mathrm{r}+\rho_\mathrm{l}] = \frac{2\Omega_\mathrm{in}}{\Gamma}\left(\frac{1}{2}+\mathrm{Re}[\rho_\mathrm{cb}]\right)\frac{1+M^2}{1+3M^2}, \\ \mathrm{Im}[\rho_\mathrm{r}-\rho_\mathrm{l}] = \frac{4\Omega_\mathrm{in}}{\Gamma}\mathrm{Im}[\rho_\mathrm{cb}]\frac{M}{1+3M^2}\cos(\omega_\mathrm{m}t). \end{cases} \tag{27}$$

By substituting Supplementary Equation 27 into Supplementary Equation 26, one has

$$S_{z,\mathrm{out}} = \frac{2\mathrm{Im}[\rho_\mathrm{cb}]\frac{M}{1+M^2}\cos(\omega_\mathrm{m}t)}{\frac{1+3M^2}{1+M^2}\frac{\Gamma}{2\varsigma L}-\left(\frac{1}{2}+\mathrm{Re}[\rho_\mathrm{cb}]\right)}. \tag{28}$$

In our experiment, the atoms are pumped into the dark state for small two-photon detuning, and consequently, $\left(\frac{1}{2}+\mathrm{Re}[\rho_\mathrm{cb}]\right) \to 0$, and $S_{z,\mathrm{out}}$ is mainly determined by $\mathrm{Im}[\rho_\mathrm{cb}]$. Then, Supplementary

Equation 28 can be simplified as $S_{z,\mathrm{out}} = 4\xi L\cos(\omega_{\mathrm{m}}t)$. As seen, in our FM experiment the major contribution in $S_{z,\mathrm{out}}$ is from the first sidebands at frequencies $\pm\omega_{\mathrm{m}}$.

Supplementary Note 6. $g^{(2)}(0)$ linewidth $\mathcal{L}$ and derivation of the optical Hamiltonian

In quantum optics, the second-order temporal correlation function between two light fields defined by Glauber has the form

$$g^{(2)}(\tau) = \frac{\langle E_1^{(-)}(t)E_2^{(-)}(t+\tau)E_2^{(+)}(t+\tau)E_1^{(+)}(t)\rangle_T}{\langle E_1^{(-)}(t)E_1^{(+)}(t)\rangle_T\langle E_2^{(-)}(t)E_2^{(+)}(t)\rangle_T}, \tag{29}$$

with the intensities $I_{\mathrm{j}}(t) = E_{\mathrm{j}}^{(-)}(t)E_{\mathrm{j}}^{(+)}(t)$ $(j = 1,2)$ and τ being the time lag between the two photodetector triggers. In this correlation-resonance spectroscopy, we are interested in $g^{(2)}(\tau)$ at the zero-time lag ($\tau = 0$),

$$g^{(2)}(0) = \frac{\langle I_1(t)I_2(t)\rangle_T}{\langle I_1(t)\rangle_T\langle I_2(t)\rangle_T}. \tag{30}$$

To use $g^{(2)}(0)$ for spectroscopy and sensing, a modified expression is used, which is bound between -1 and 1:

$$g^{(2)}_{\mathrm{WM}}(0) = \frac{\langle I_1(t)I_2(t)\rangle_T}{\sqrt{\langle I_1^2(t)\rangle_T\langle I_2^2(t)\rangle_T}}. \tag{31}$$

In addition, here $I_1(t)$ and $I_2(t)$ are the intensities after the frequency post-selection, as shown by Supplementary Equation 14. Simple calculations give

$$\begin{cases} I_1(t) = I_{\mathrm{r}}(t) - D\langle I_{\mathrm{r}}(t)\rangle_T\,, \\ I_2(t) = I_{\mathrm{l}}(t) - D\langle I_{\mathrm{l}}(t)\rangle_T\,, \end{cases} \tag{32}$$

where $I_{\mathrm{r}}(t)$ and $I_{\mathrm{l}}(t)$ are respectively the recorded intensities for the right-circular polarization and left-circular polarization subject to the WM procedure. For simplicity, however, we will omit the subscript "WM" in all $g^{(2)}_{\mathrm{WM}}(0)$ expressions hereafter in this paper.

According to previous results, when the two-photon detuning $\Delta = 0$, the light transmittance for left-circular and right-circular polarizations is perfectly synchronized and yields the same values when varying the one-photon detuning δ. In this case, $g^{(2)}(0) = 1$. However, when $\Delta \neq 0$, because of the breach of symmetry between left- and right-circular polarization, $g^{(2)}(0)$ will decrease and become -1 for large Δ. Since Δ is introduced by an externally applied magnetic field *B* along the light propagation direction, the change of $g^{(2)}(0)$ from 1 to -1 in turn reflects the change of *B*. The linewidth $\mathcal{L}$ of $g^{(2)}(0)$ therefore marks the ability of magnetic field sensing.

Since the $g^{(2)}(0)$ value has the bound $[-1,1]$, $\mathcal{L}$ can be obtained from solving $g^{(2)}(0) = 0$. As such, to theoretically identify $\mathcal{L}$, we begin with the numerator in Supplementary Equation 31,

$$\langle I_1(t)I_2(t)\rangle_T = \frac{1}{4}\langle[I_1(t) + I_2(t)]^2 - [I_1(t) - I_2(t)]^2\rangle_T. \tag{33}$$

By using Supplementary Equation 32, the first term on the right hand side becomes $\langle [I_1(t) + I_2(t)]^2 \rangle_T \approx [(1-D)\langle I_{\mathrm{r}}(t) + I_{\mathrm{l}}(t)\rangle_T]^2$, and the second term becomes $\langle [I_1(t) - I_2(t)]^2 \rangle_T \approx [\langle I_{\mathrm{r}}(t) - I_{\mathrm{l}}(t)\rangle_T]^2$.
With these preparations, we have:

$$g^{(2)}(0) = 0 \Longrightarrow \langle I_1(t) I_2(t)\rangle_T = 0 \Longrightarrow 1 - \frac{\langle [I_1(t)-I_2(t)]^2\rangle_T}{\langle [I_1(t)+I_2(t)]^2\rangle_T} = 0 \Rightarrow 1 - \langle \left[\frac{I_1(t)-I_2(t)}{\langle I_1(t)+I_2(t)\rangle_T}\right]^2 \rangle_T = 0. \tag{34}$$

Interestingly, we note that the argument in Supplementary Equation 34 is related to S_z,

$$\frac{I_1(t)-I_2(t)}{\langle I_1(t)+I_2(t)\rangle_T} = S_z. \tag{35}$$

With the help of Supplementary Equations 23, 34 and 35, Supplementary Equation 34 yields

$$\langle \sigma_z \rangle_{\mathrm{pf}} = \sqrt{2}. \tag{36}$$

In addition, the $g^{(2)}(0)$ linewidth, $\mathcal{L}$, is quantitatively characterized by

$$\mathcal{L} = \frac{\sqrt{2}}{\left| \partial \langle \sigma_z \rangle_{\mathrm{pf}} / \partial \Delta \right|}. \tag{37}$$

Now we are ready to interpret the physics using the optical Hamiltonian and weak value. As the measurement is implemented on light intensities $\left(\propto |\Omega_{\mathrm{outr(l)}}|^2\right)$, only the imaginary parts of the atomic optical coherences $\rho_{\mathrm{r(l)}}$ are of importance, i.e.,

$$\mathrm{Im}[\rho_{\mathrm{r(l)}}(t)] = \frac{2M}{1+3M^2} \frac{\Omega_{\mathrm{in}} \mathrm{Im}(\rho^{(0)}_{\mathrm{cb(bc)}})}{\Gamma} \cos(\omega_{\mathrm{m}} t). \tag{38}$$

Here,

$$\cos(\omega_{\mathrm{m}} t) = \frac{e^{-i\omega_{\mathrm{m}} t} + e^{i\omega_{\mathrm{m}} t}}{2} = \frac{|-\omega_{\mathrm{m}}\rangle\langle 0| + |\omega_{\mathrm{m}}\rangle\langle 0|}{2}. \tag{39}$$

Now the effective Hamiltonians $-\kappa_{\mathrm{r(l)}}$ can be cast together in a compact form,

$$H = -i \frac{\varsigma}{\Gamma} \frac{M}{1+3M^2} \mathrm{Im}[\rho_{\mathrm{cb}}] \begin{pmatrix} 1 & 0 \\ 0 & -1 \end{pmatrix} \otimes (|-\omega_{\mathrm{m}}\rangle\langle 0| + |\omega_{\mathrm{m}}\rangle\langle 0|). \tag{40}$$

By using the result in Supplementary Equation 17, Supplementary Equation 40 takes the final form

$$H = -i\xi \boldsymbol{\sigma}_z \otimes (|-\omega_{\mathrm{m}}\rangle\langle 0| + |\omega_{\mathrm{m}}\rangle\langle 0|), \tag{41}$$

where the grouped constant $\xi = \frac{\varsigma}{\Gamma} \frac{M}{1+3M^2} \mathrm{Im}[\rho_{\mathrm{cb}}]$ represents the effective interaction strength between the system and the pointer. In Supplementary Equation 41, the pointer operator $\boldsymbol{\sigma}_z = \begin{pmatrix} 1 & 0 \\ 0 & -1 \end{pmatrix}$, by acting on the corresponding Jones vectors, will produce the meaningful observable (the Stokes parameter S_z), to characterize the polarization change due to the absorption difference between the two atomic transitions, $|b\rangle \to |a\rangle$ and $|c\rangle \to |a\rangle$. Due to the absorption nature of the

atom-light interaction, the effective Hamiltonian Supplementary Equation 41, as well as Supplementary Equation 40 is purely imaginary and non-Hermitian. The system operator $(|-\omega_{\mathrm{m}}\rangle\langle 0| + |\omega_{\mathrm{m}}\rangle\langle 0|)$ accounts for the FM to AM conversion process that generates the first order sidebands frequency component in the rotating frame of the frequency-modulated laser. Let's recall that in the Jones vector representation, the full polarization information of light has to resort to both the polarization vector and the frequency vector in general, because they are correlated.

Supplementary Note 7. Weak value and its induced linewidth reduction

Given the effective Hamiltonian (Supplementary Equation 41), we are now ready to look at the weak value. With the pre- and post-selected frequency states shown in Supplementary Equation 13 and Supplementary Equation 14, respectively, the final polarization of the output fields evolves to

$$
\begin{aligned}
&|\Phi_{\mathrm{pf}}\rangle' \\
&= \langle\Psi_{\mathrm{sf}}|e^{-iHL}|\Phi_{\mathrm{pi}}\rangle \otimes |\Psi_{\mathrm{si}}\rangle \\
&\simeq \langle\Psi_{\mathrm{sf}}|\Psi_{\mathrm{si}}\rangle|\Phi_{\mathrm{pi}}\rangle - iL\langle\Psi_{\mathrm{sf}}|H|\Phi_{\mathrm{pi}}\rangle \otimes |\Psi_{\mathrm{si}}\rangle \\
&= \langle\Psi_{\mathrm{sf}}|\Psi_{\mathrm{si}}\rangle|\Phi_{\mathrm{pi}}\rangle - i\xi L\boldsymbol{\sigma}_z|\Phi_{\mathrm{pi}}\rangle\langle\Psi_{\mathrm{sf}}|(|-\omega_{\mathrm{m}}\rangle\langle 0| + |\omega_{\mathrm{m}}\rangle\langle 0|)|\Psi_{\mathrm{si}}\rangle,
\end{aligned}
\tag{42}
$$

The formal normalization of Supplementary Equation 42 gives

$$
|\Phi_{\mathrm{pf}}\rangle = (1 - \xi L A_{\mathrm{W}}\boldsymbol{\sigma}_z)|\Phi_{\mathrm{pi}}\rangle \simeq e^{-\xi L A_{\mathrm{W}}\boldsymbol{\sigma}_z}|\Phi_{\mathrm{pi}}\rangle,
\tag{43}
$$

where the weak value A_{W} (originally defined by Aharonov, Albert, and Vaidman) is real and takes the value of

$$
A_{\mathrm{W}} = \frac{\langle\Psi_{\mathrm{sf}}|(|-\omega_{\mathrm{m}}\rangle\langle 0|+|\omega_{\mathrm{m}}\rangle\langle 0|)|\Psi_{\mathrm{si}}\rangle}{\langle\Psi_{\mathrm{sf}}|\Psi_{\mathrm{si}}\rangle} = \frac{2}{1-D}.
\tag{44}
$$

With $|\Phi_{\mathrm{pf}}\rangle$ given in Supplementary Equation 43, the output polarization can be computed as

$$
\langle\sigma_z\rangle_{\mathrm{pf}} = \langle\Phi_{\mathrm{pf}}|\boldsymbol{\sigma}_z|\Phi_{\mathrm{pf}}\rangle = -2\xi L A_{\mathrm{W}}\,,
\tag{45}
$$

signalling the anomalous amplification of $\langle\sigma_z\rangle$ outside of the conventional inequality $|\langle\sigma_z\rangle| \leq 1$. With the use of Supplementary Equations 6, 7, 35, 37, and 45, the linewidth $\mathcal{L}$ now assumes the expression

$$
\mathcal{L} = \frac{1}{\sqrt{2}\left|LA_{\mathrm{W}}\left(\frac{\partial\xi}{\partial\Delta}\right)\right|} = \frac{\Gamma}{2\sqrt{2}\varsigma L}\frac{((1+3M^2)\gamma_2+2(1+M^2)\Gamma_{\mathrm{p0}})^2}{M(1+M^2)\Gamma_{\mathrm{p0}}}\frac{1}{A_{\mathrm{W}}}\,,
\tag{46}
$$

clearly indicating that the anomalous WV amplification results in the narrowing of the correlation-resonance linewidth.

Supplementary Note 8. Magnetometer Sensitivity

In our scheme, near $g^{(2)}(0) = 0$ of a Lorentzian resonance profile, the magnetic field sensitivity can be simply calculated by

$$\mathbb{S} = \frac{2\mathcal{L}}{\mathrm{SNR}} \tag{47}$$

where SNR is the signal-to-noise ratio of the $g^{(2)}(0)$ correlation resonance, with the signal amplitude equal to 2 and the noise amplitude equal to $\delta g^{(2)}(0)$. For experiments without any technical noises, we can obtain the photon-shot-noise limited SNR:

$$\mathrm{SNR} = \sqrt{2}(1-D)\sqrt{n_{\mathrm{ph}}} = \frac{2\sqrt{2}}{A_{\mathrm{W}}}\sqrt{n_{\mathrm{ph}}}, \tag{48}$$

where n_{ph} is the photon-number rate. Conversely, SNR degrades by the same factor A_{W} by which the linewidth is narrowed, rendering a sensitivity independent of WV. However, as shown in the following section, in the presence of technical noise, the sensitivity can be optimized by choosing the proper post-selection parameter in the WM process.

Supplementary Note 9. Effects of the residual amplitude modulation (RAM) on the sensitivity

Under the assumption of unit quantum efficiency for the two photodetectors, the two recorded signals can be written as

$$\begin{cases} I_{\mathrm{r}} = \eta + \sqrt{2}\alpha\cos(\omega_{\mathrm{m}}t) + \sqrt{2}\,\langle\eta\rangle\,\mathrm{RAM}\cos\big(\omega_{\mathrm{m}}t + \varphi(t)\big) \\ I_{\mathrm{l}} = \eta' - \sqrt{2}\alpha'\cos(\omega_{\mathrm{m}}t) + \sqrt{2}\,\langle\eta\rangle\,\mathrm{RAM}\cos\big(\omega_{\mathrm{m}}t + \varphi(t)\big) \end{cases}, \tag{49}$$

where η and η' are the DC parts of the signals, α and α' the AC parts, and RAM represents the percentage noise amplitude of the residual amplitude modulation. Since the photon number satisfies a Poisson distribution, we have $\langle\eta^2\rangle = \langle\eta\rangle^2 + \langle\eta\rangle$. In the experiment, the DC part becomes $\langle\xi\rangle = \langle\xi'\rangle = (1-\mathrm{D})\langle\eta\rangle$ after post-selection, and $\langle\xi^2\rangle = \langle\xi'^2\rangle = ((1-\mathrm{D})\langle\eta\rangle)^2 + \langle\eta\rangle$. The signals can be rewritten as

$$\begin{cases} I_1 = \xi + \sqrt{2}\alpha\cos(\omega_{\mathrm{m}}t) + \sqrt{2}\,\langle\eta\rangle\,\mathrm{RAM}\cos\big(\omega_{\mathrm{m}}t + \varphi(t)\big) \\ I_2 = \xi' - \sqrt{2}\alpha'\cos(\omega_{\mathrm{m}}t) + \sqrt{2}\,\langle\eta\rangle\,\mathrm{RAM}\cos\big(\omega_{\mathrm{m}}t + \varphi(t)\big) \end{cases}. \tag{50}$$

With the definitions $S_{12} = \langle I_1 I_2\rangle_t = \langle\xi\xi'\rangle - \langle\alpha\alpha'\rangle + \langle\eta\rangle^2\mathrm{RAM}^2$, $S_{11} = \langle I_1 I_1\rangle_t = \langle\xi^2\rangle + \langle\alpha^2\rangle + \langle\eta\rangle^2\mathrm{RAM}^2$ and $S_{22} = \langle I_2 I_2\rangle_t = \langle\xi'^2\rangle + \langle\alpha'^2\rangle + \langle\eta\rangle^2\mathrm{RAM}^2$, $g^{(2)}(0)$ can be expressed as $g^{(2)}(0) = \frac{S_{12}}{S_{11}}$ for $S_{11} = S_{22}$ (which is the case here). The noise on the $g^{(2)}(0)$ value is

$$\delta g^{(2)}(0) = \frac{\delta S_{12}}{S_{11}} + \frac{S_{12}\delta S_{11}}{{S_{11}}^2} = \frac{\sqrt{1+2\left(g^{(2)}(0)\right)^2}(1+g^{(2)}(0))}{(1-D)\sqrt{n_{\mathrm{ph}}/2}}\sqrt{\frac{1}{1+g^{(2)}(0)} + \frac{\delta^2\mathrm{RAM}}{2} + \frac{6\mathrm{RAM}^2}{(1-D)^2}}. \tag{51}$$

Under the condition that $\mathrm{RAM} \ll \alpha$, the practical sensitivity now takes the form

$$\mathbb{S} = \frac{\sqrt{1+2(g^{(2)}(0))^2}}{\sqrt{1-(g^{(2)}(0))^2}} \frac{\mathcal{L}}{(1-D)\sqrt{n_{\mathrm{ph}}/2}} \sqrt{\frac{1}{1+g^{(2)}(0)} + \frac{\delta^2\mathrm{RAM}}{2} + \frac{6\mathrm{RAM}^2}{(1-D)^2}}, \quad (52)$$

where $\delta^2\mathrm{RAM} \equiv \langle \mathrm{RAM}^2 \rangle - \langle \mathrm{RAM} \rangle^2$ is a small fluctuation.

From Supplementary Equation 52, one can see that, for a certain amount of RAM, the practical sensitivity can be optimized by choosing a suitable *D* value, as illustrated in Supplementary Figure 1. The practical sensitivity improves for smaller *D* values and then stays nearly constant below a certain *D* value. This predicted trend agrees qualitatively with the experiment, as shown in Fig. 4 in the main text. In practice, however, the sensitivity becomes slightly worse when *D* is smaller because the linewidth in this region is beyond the small two-photon detuning approximation (i.e., assuming that $\mathrm{Im}[\rho_{\mathrm{cb}}]$ is linear with *B*) in the simplified model. Additionally, a similar amount of degradation in sensitivity requires less RAM than that calculated in the theory, which is likely due to the absence of other noise sources in the model, including the magnetic field noise.

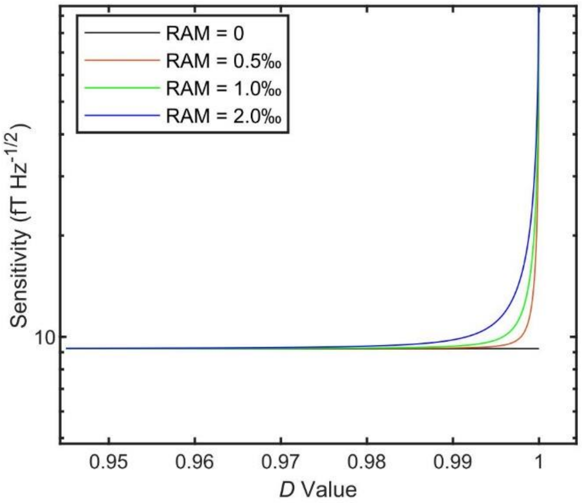

Supplementary Figure 1 | Practical sensitivity calculated in the presence of laser intensity noise. For different amounts of residual amplitude modulation (RAM), the theory shows that the optimal sensitivity is achievable by choosing a proper *D* value. The parameters involved in the numerical simulations are the same as those used in Fig. 4 in the main text.

Supplementary Note 10. Bandwidth of the magnetometer

The bandwidth of the magnetometer is associated with the ground state dynamics of the atoms and is mainly determined by the optical pumping process establishing the dark state for EIT. In Fig. 3b in the main text, the sensitivity spectrum is obtained from the $g^{(2)}(0)$ noise spectrum and the resonance profile's slope at different magnetic field frequencies. To compute the slope, i.e., the response curve, we set the two-photon detuning (via the applied magnetic field) to be $\Delta = \Delta_0 + \Delta_1 \sin(\omega_{\mathrm{B}} t)$, where Δ_0 is

the HWHM of the $g^{(2)}(0)$ resonance, and Δ_1 is set to be small to remain in the linear region of the slope. Then, we numerically solve the master equation to obtain the transmission signal, from which the oscillation amplitude (A) of $g^{(2)}(0)$ and the slope A/Δ_1 can be sequentially obtained. As shown in Supplementary Figure 2, the calculated response can be well fitted by the function $\mathrm{BW}/\sqrt{\mathrm{BW}^2 + f^2}$, with a BW value of 9.2 Hz, giving an HWHM of approximately 16 Hz, close to the power broadened EIT linewidth in the theoretical model. In the calculation of this response curve, no noise has been included. In fact, the presence of noise can affect the linewidth of this curve. For instance, if the noise level is higher (hence a broader $g^{(2)}(0)$ resonance and a smaller slope) at a lower frequency than at higher frequency, such as that from the $1/f$ noise, the measured response will be slightly broadened, resulting in a broader response curve than what was calculated, as shown in Fig. 3b in the main text.

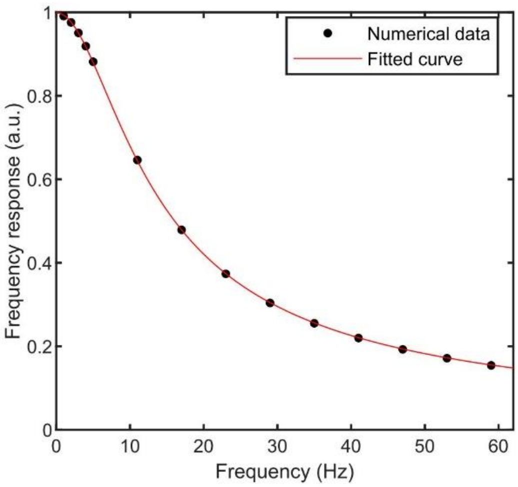

Supplementary Figure 2 | Calculated frequency response of the magnetometer. The curve well matches the function $\mathrm{BW}/\sqrt{\mathrm{BW}^2 + f^2}$ with the parameter BW=9.2 Hz.

In this work, we found our magnetometer still offers an appreciable sensitivity, even in the frequency range beyond 60 Hz. As an example, Supplementary Figure 3 reports the typical experimental results of the magnetometer sensitivity for the range from near DC up to 200 Hz. As one can see, in the range of 2 – 100 Hz the measured sensitivity can remain below 20 fT Hz$^{-1/2}$. Although, along with the growth in the frequency, the sensitivity becomes worse. However, even near 200 Hz, the sensitivity only drops to approximately 40 fT Hz$^{-1/2}$.

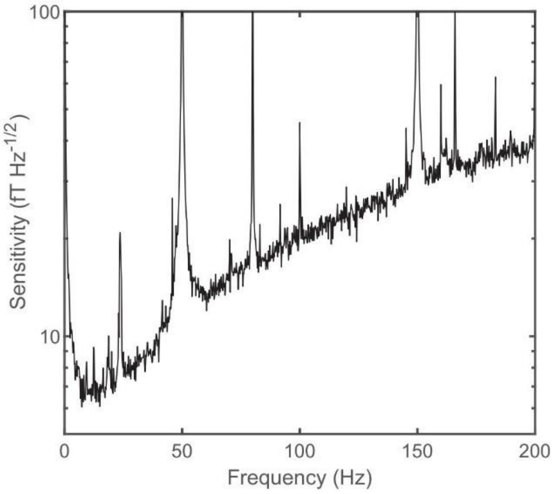

Supplementary Figure 3 | Magnetic field sensitivity from near DC to 200 Hz.

Supplementary Note 11. Characterization of the coherence-lifetime-limited linewidth of EIT

The normal EIT resonance has a linewidth that is composed of two parts [2]: the coherence lifetime limited linewidth, and the power broadened linewidth. The former can be found by the extracted zero-power EIT linewidth, as shown in Supplementary Figure 4. We found that the HWHM zero-power width is 0.76 Hz, which is mainly determined by the residual magnetic field inhomogeneity in the vapor cell and is much larger than that limited by the quality of the anti-relaxation coating material on the cell wall.

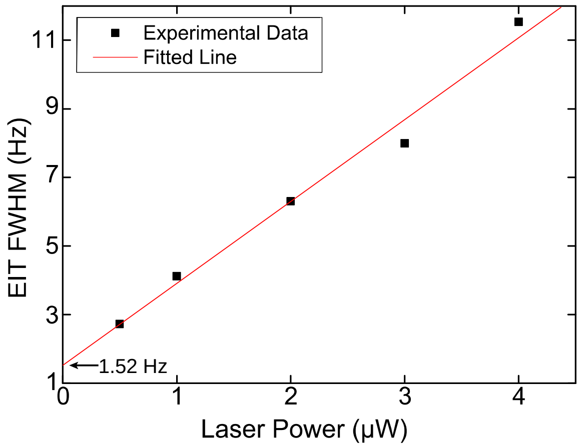

Supplementary Figure 4 | Measured EIT linewidth (full width at half maximum) vs. input laser power. The EIT linewidth has a linear dependence on the laser power for a relatively low power. The linear fit gives the "zero-power" linewidth, which is essentially determined by the lifetime of the ground state coherence.

The conventional NMOR magnetometer [3] uses the same atom-light interaction process as EIT, and its linewidth is the same as that of the EIT measured here. The only difference is that, here in the EIT experiment, the total laser power at the cell output is measured, while in NMOR the polarization rotation of the laser field is measured.

Supplementary References

1. Spreeuw, R. J. C. A classical analogy of entanglement. *Found. Phys.* **28**, 361-374 (1998).
2. Xiao, Y. Spectral line narrowing in electromagnetically induced transparency. *Mod. Phys. Lett. B.* **23**, 661-680 (2009).
3. Budker, D., Gawlik, W., Kimball, D. F., Rochester, S.M., Yashchuk, V. V., & Weis A. Resonant nonlinear magneto-optical effects in atoms. *Rev. Mod. Phys.* **74**, 1153-1201 (2002).
